# Trophic specialisation reflected by radular tooth material properties in an “ancient” Lake Tanganyikan gastropod species flock

**DOI:** 10.1186/s12862-021-01754-4

**Published:** 2021-03-03

**Authors:** Wencke Krings, Marco T. Neiber, Alexander Kovalev, Stanislav N. Gorb, Matthias Glaubrecht

**Affiliations:** 1 Center of Natural History (CeNak), Universität Hamburg, Martin-Luther-King-Platz 3, 20146 Hamburg, Germany; 2 Zoological Institute of the Christian-Albrechts-Universität zu Kiel, Am Botanischen Garten 9, 24118 Kiel, Germany

**Keywords:** Functional morphology, Nanoindentation, Mechanical properties, Gastropoda, Trophic specialisation, Adaptive radiation

## Abstract

**Background:**

Lake Tanganyika belongs to the East African Great Lakes and is well known for harbouring a high proportion of endemic and morphologically distinct genera, in cichlids but also in paludomid gastropods. With about 50 species these snails form a flock of high interest because of its diversity, the question of its origin and the evolutionary processes that might have resulted in its elevated amount of taxa. While earlier debates centred on these paludomids to be a result of an intralacustrine adaptive radiation, there are strong indications for the existence of several lineages before the lake formation. To evaluate hypotheses on the evolution and radiation the detection of actual adaptations is however crucial. Since the Tanganyikan gastropods show distinct radular tooth morphologies hypotheses about potential trophic specializations are at hand.

**Results:**

Here, based on a phylogenetic tree of the paludomid species from Lake Tanganyika and adjacent river systems, the mechanical properties of their teeth were evaluated by nanoindentation, a method measuring the hardness and elasticity of a structure, and related with the gastropods’ specific feeding substrate (soft, solid, mixed). Results identify mechanical adaptations in the tooth cusps to the substrate and, with reference to the tooth morphology, assign distinct functions (scratching or gathering) to tooth types. Analysing pure tooth morphology does not consistently reflect ecological specializations, but the mechanical properties allow the determination of eco-morphotypes.

**Conclusion:**

In almost every lineage we discovered adaptations to different substrates, leading to the hypothesis that one main engine of the flock’s evolution is trophic specialization, establishing distinct ecological niches and allowing the coexistence of taxa.

## Background

Hypotheses on how biodiversity relates with the temporal and spatial “filling” of available habitats and annidation itself, i.e. the actual formation of ecological niches as a combined process of internal and external factors, is paramount for understanding how species evolve under geographical and ecological conditions [1–3]. Addressing this topic has seen various approaches based on vertebrates, such as the studies on Darwin finches [4–9], the cichlid fishes in the East African lakes [10–13] and Nicaragua [14–16] or *Anolis* lizards [17–19]. These species flocks [cf. 20] are usually regarded as examples of adaptive radiations, the evolution of ecological and phenotypic diversity within a rapidly multiplying lineage, thus linking speciation and ecology [21–26].

**Fig. 1:**
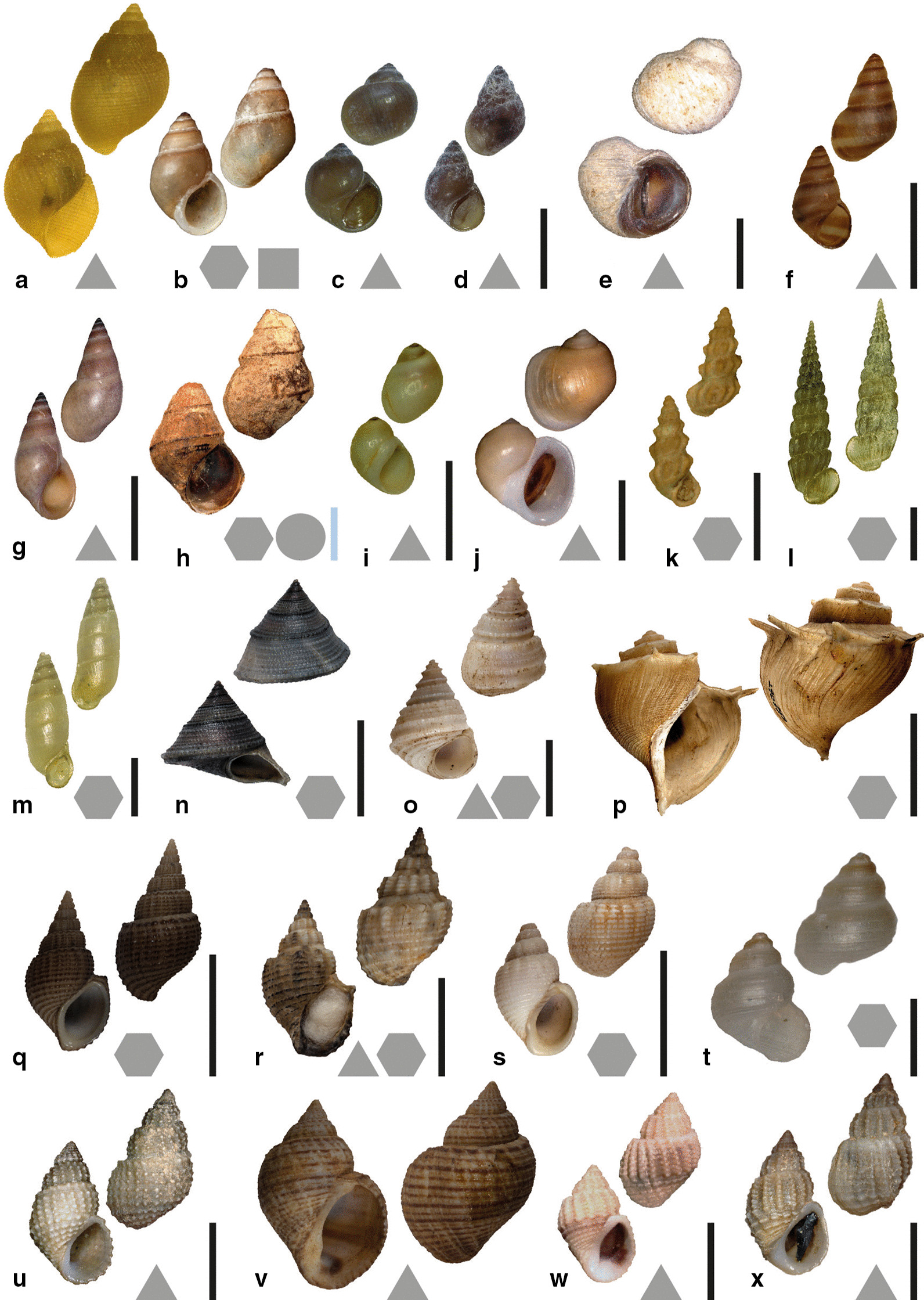
Shells of examined species. Black scale bars: from Lake Tanganyika. Blue scale bar: from adjacent river systems. Forms next to the letters **a**–**x** indicate preferred feeding substrate (circle = mud, hexagon = sand, square = plants, triangle = rock). **a** *Bridouxia ponsonbyi* ZMB 220.137-1, **b** *B. grandidieriana* BMNH 1889.6.23.57-61, **c** *B. rotundata* ZMB 220.063-3, **d** *B. praeclara* DBL 19-4, **e** *Spekia zonata* ZMB 220.007-2, **f** *Leloupiella minima* ZMB 220.008-3, **g** *Reymondia horei* ZMB 220.007-1, **h** *Cleopatra johnstoni* ZMB 220.102, **i** *Stanleya neritinoides* ZMB 102.624-1, **j** *Tanganycia rufofilosa *ZMB 102.621-1, **k** *Martelia tanganyicensis* ZMB 220.134-1, **l** *Anceya giraudi* ZMB 220.000-1, **m** *Syrnolopsis lacustris* ZMB 220.046-1, **n** *Chytra kirki* SMF 290543-1, **o** *Limnotrochus thomsoni* SMF 290542-1, **p** *Tiphobia horei* SMF 290550-1, **q** *Paramelania iridescens* SMF 290,538, **r** *P. damoni* SMF 290531-2, **s** *P. crassigranulata* SMF 290528-1, **t** *Mysorelloides multisulcata* DBL without number, **u** *Lavigeria spinulosa* ZMB 220114, **v** *L. grandis* SMF 292827-1, **w** *L. nassa* ZMB 220.172-1, **x** *L. livingstoniana* ZMB 220116; Scale bars: **a**–**d**, **f**, **k**, **l**, **m** = 2.5 mm; **e** = 5 mm; **g**, **i**, **j**, **n**, **o**, **v**, **w** = 10 mm; **h** = 5 mm; **p**–**s** = 20 mm, **t** = 0.75 mm; **u**, **x** = 6 mm

However, even though the majority of all known animals are invertebrates [27, 28], fewer model systems were on focus (with exceptions, e.g. [29, 30] on crickets). There are however spectacular examples of invertebrate species flocks exhibiting a great diversity, especially among molluscs [see also 31, 32], the lacustrine and riverine freshwater gastropods on Sulawesi [33–38], Madagascar [39], in the Thai rivers [40, 41], or “ancient” Lake Tanganyika [42–44]. The latter is well known for its unique assemblage of endemic species and has been a natural laboratory for research on the drivers of evolution for decades. It harbours paludomid gastropods (Fig. 1) which triggered many expeditions and subsequently malacological descriptions [e.g. 45–60] and a long-lasting controversy about the origin and evolution of the lake and its fauna. Due to their marine-like appearance (termed “thalassoid” by [45] and “halolimnic” by [61]) many earlier authors addressed the possibility of a marine origin of the Lake Tanganyika fauna and discussed the causes of the thalassoid appearance of its endemic molluscs. However, this eventually led to the refutation of Moore’s controversial hypothesis [61–63] of the lake being once directly connected to the ocean [see e.g. in 64–68].

For a long time the idea that this largest and deepest of the African lakes has supplied its gastropod diversity with a stable inland environment and offered unique opportunities for within-lake diversification (“ancient endemic radiation” see [65, 69]) resulting in a truly “adaptive” radiation [cf. 70, 71] was common [e.g. 72–86]. However, strong evidence for an ancient origin of disparity and diversity in this flock has been presented, indicating the existence of major gastropod lineages before the formation of the lake itself or its proto-lakes. The oldest formation estimates are 9–12 Mya [87–89], while more recent studies date the pre-rift formation to 4–11 Mya and the earliest onset of a true rifting activity to 5.5 Mya [90–93]. With a molecular clock approach in support [42], this alternative hypothesis of the former existence of several originally riverine paludomid lineages later inhabiting the lake and bringing possible adaptations to former river environments with them was suggested by [43, 44].

To allocate hypotheses about paludomid evolution and radiations—especially in the context of adaptive radiations—the identification of actual adaptation is crucial. Morphological structures associated with feeding, such as e.g. bills or skulls in birds, vomer bones in cichlid fishes or teeth in mammals, can exhibit adaptations and indicate trophic specialization. They serve as an interface between the organism and its ingesta (food, minerals) and, as they provided insights into functional adaptations and hence evolution, are of high research interest in various groups [e.g. 94–97 on Darwin’s finches, 98 on oviraptorosaurian dinosaurs, 99 on cichlid fish].

**Fig. 2:**
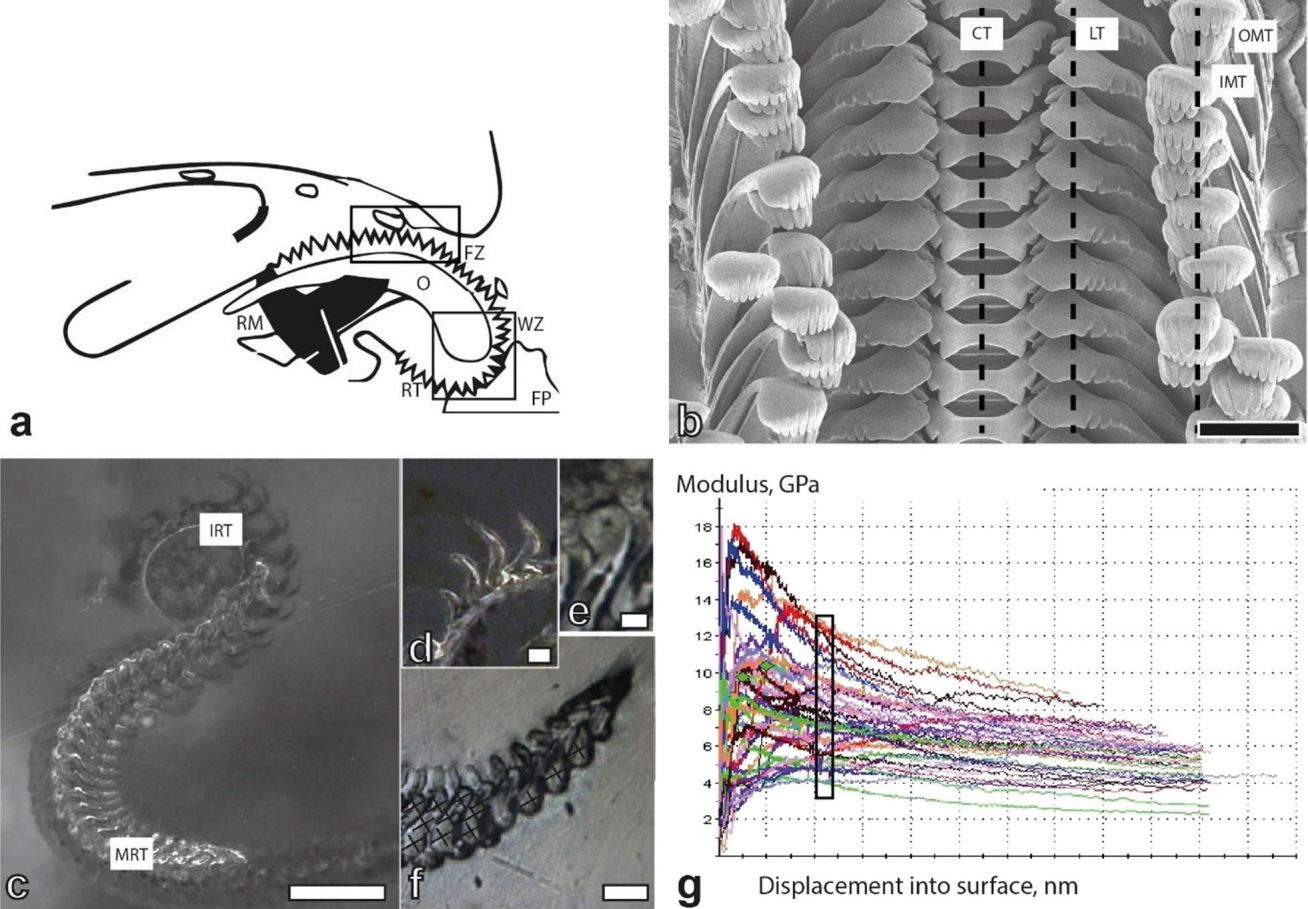
**a** Schematic drawing of the radula when feeding, **b** Taenioglossan radula of *Spekia zonata* (ZMH 150008/999-2), black line = area of nanoindentation for central, lateral, and marginal teeth, **c** Radula (ZMB220.143-2) embedded in epoxy resin and polished for nanoindentation (longitudinal section along the radula) with (**d**–**f**) magnification of some tested areas (**e** with nanoindentation mark; **f** crosses indicate points of indentation), **g** Representative results as nanoindentation measurement curves for basis, stylus, and cusps of central teeth (Young’s modulus, GPa, versus displacement into tooth material). The values for the cusps within the indentation depth of 480–520 nm were used for further calculation. Scale bars: **b** = 100 µm; **c** = 250 µm; **d**, **e** = 30 µm; **f** = 60 µm. *CT* central tooth, *FP* food particle, *FZ* formation zone, *IMT* inner marginal tooth, *IRT* immature radular teeth, *LT* lateral tooth, *MRT* mature radular teeth, *O* odontophore, *OMT* outer marginal tooth, *RM* radular muscles, *RT* radular teeth, *WZ* working zone

The gastropods radula, one important synapomorphy of the Mollusca, acts as such an interface, mechanically processing ingesta and directly linking the organism with its food. Various muscles control the motion of this feeding organ, pulling the chitinous radular membrane with rows of small embedded radular teeth [100] across an odontophoral cartilage (Fig. 2a). As teeth are in direct contact with the ingesta, their morphology can often be directly linked with the animal’s ecology and can reflect various transitions from zoovorous to herbivorous traits [101–106]. Form together with the tooth’s position and chemical composition are widely considered adaptive to food and are hence closely associated with feeding strategies, competitor avoidance and thus trophic specialization [107–118].

Strong indications for trophic specialization in the context of gastropod adaptive radiations have been described for the radular tooth morphologies of lacustrine *Tylomelania* from Sulawesi [33, 35, 38], the riverine gastropods from Kaek River [40], and marine *Dendronotus* [119]. For the Lake Tanganyikan paludomids hypotheses on the potential influence of trophic specialization on the evolution of this flock are consequential, since these species show an extraordinarily high interspecific diversity in tooth morphologies [e.g. 44, 120]. These shapes can often be related with the gastropods’ specific feeding substrates (soft, mixed or solid) since teeth as highly functionate interfaces do not only interact with the food but also with the substrate the food is attached to [121, 122]. In addition, recent studies on the paludomid tooth anchorages in the radular membrane, which are also diverse between taxa, relate this connecting area with the gastropod’s specific feeding substrate [123].

In addition to morphology, the structural composition also influences functionality. For reconstructing the evolutionary history of the African Paludomidae and to develop hypotheses on the role of trophic specialisation in their evolution, we here identified the hardness and elasticity of taenioglossan radular teeth from 24 species belonging to this flock utilising nanoindentation, a technique previously employed to identify local mechanical properties of various biological materials [e.g. 124–130]. Results, with reference to morphology, allow the assignment of distinct functions to certain tooth types. The identification of mechanical adaptations in their cusps to the preferred feeding substrate allowed the establishment of eco-morphotypes. Our results strongly indicate that one main engine of the flock’s evolution is trophic specialization, allowing the coexistence of species.

## Results

**Fig. 3:**
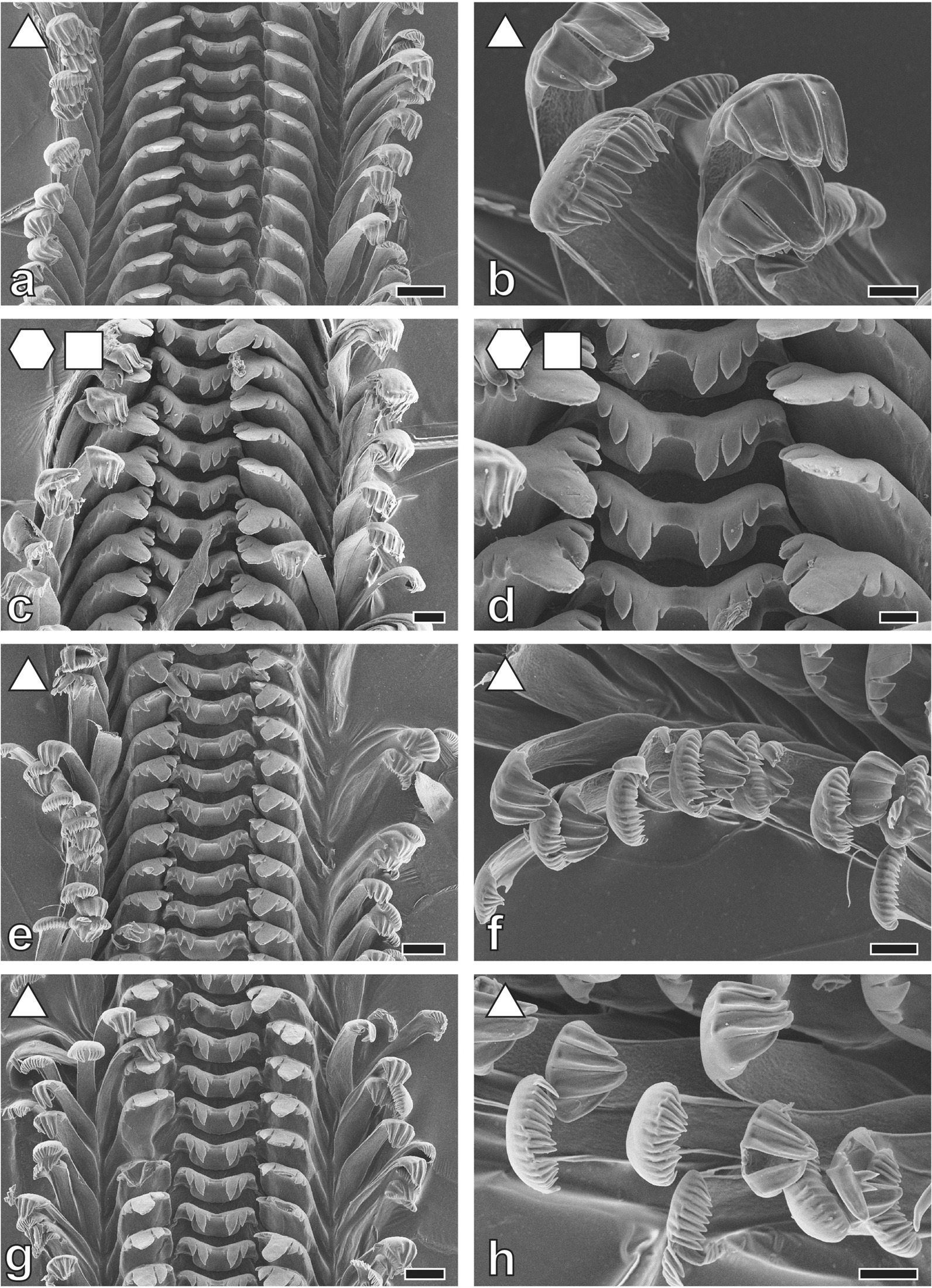
Radular teeth of: **a**, **b**  *Bridouxia ponsonbyi *ZMB 220.137-1, **a** overview, **b** marginals; **c**, **d** *B. grandidieriana* ZMB 220.139-4, **c** overview, **d** centrals and laterals; **e**, **f** *B. rotundata* ZMB 220063-1, **e** overview, **f** marginals; **g**, **h** *B. praeclara *ZMB 220.063-2, **g** overview, **h** marginals. Scale bars: **a** = 40 μm; **b**, **d**, **f**, **h** = 10 μm; **c**, **e**, **g** = 20 μm. Forms indicate preferred feeding substrate (circle = mud, hexagon = sand, square = plants, triangle = rock)

**Fig. 4:**
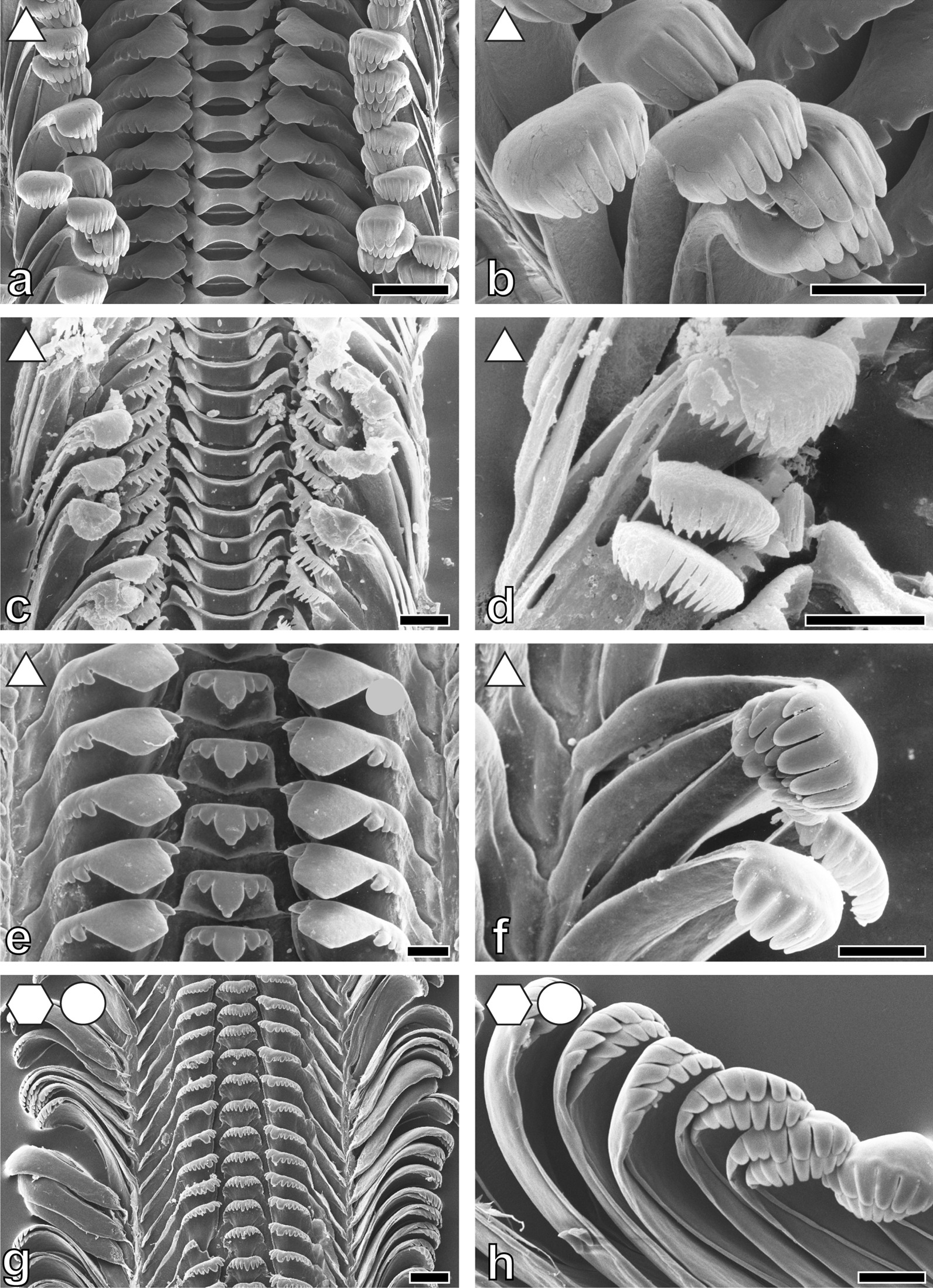
Radular teeth of: **a**, **b** *Spekia zonata* ZMH 150008/999-2, **a** overview, **b** marginals; **c**, **d** *Leloupiella minima* ZMB 220.135, **c** overview, **d** marginals; **e**, **f** *Reymondia horei *ZMB 220.147-1, **e** centrals and laterals, **f** marginals; **g**, **h** *Cleopatra* *johnstoni* ZMB 220.102-1, **g** overview, **h** marginals. Scale bars: **a** = 100 μm; **b** = 50 μm; **c**, **d** = 10 μm; **e**, **f** = 30 μm; **g** = 40 μm; **h** = 20 μm. Forms indicate preferred feeding substrate (circle = mud, hexagon = sand, square = plants, triangle = rock)

**Fig. 5:**
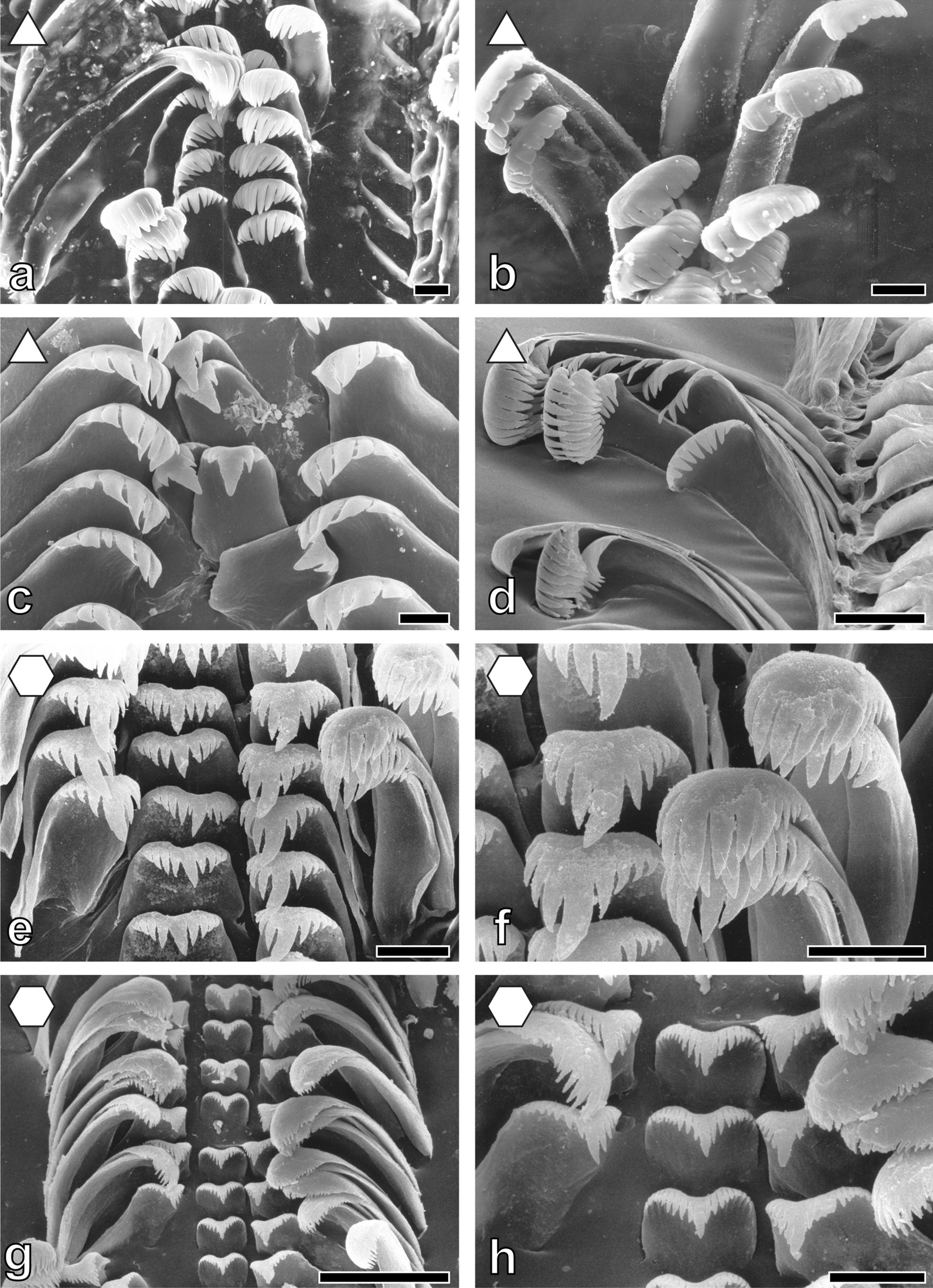
Radular teeth of: **a**, **b** *Stanleya* *neritinoides* MRAC without number, **a** centrals and laterals, **b** marginals; **c**, **d** *Tanganycia rufofilosa*, **c** centrals and laterals, **d** marginals; **e**, **f** *Martelia* *tanganyicensis* ZMB 220.133-1, **e** overview, **f** laterals and marginals; **g**, **h.** *Anceya* *giraudi* ZMB 220.132, **g** overview, **h** centrals and laterals. Scale bars: **a**–**c**, **e**, **f**, **h** = 10 μm; **d**, **g** = 30 μm. Forms indicate preferred feeding substrate (circle = mud, hexagon = sand, square = plants, triangle = rock)

**Fig. 6:**
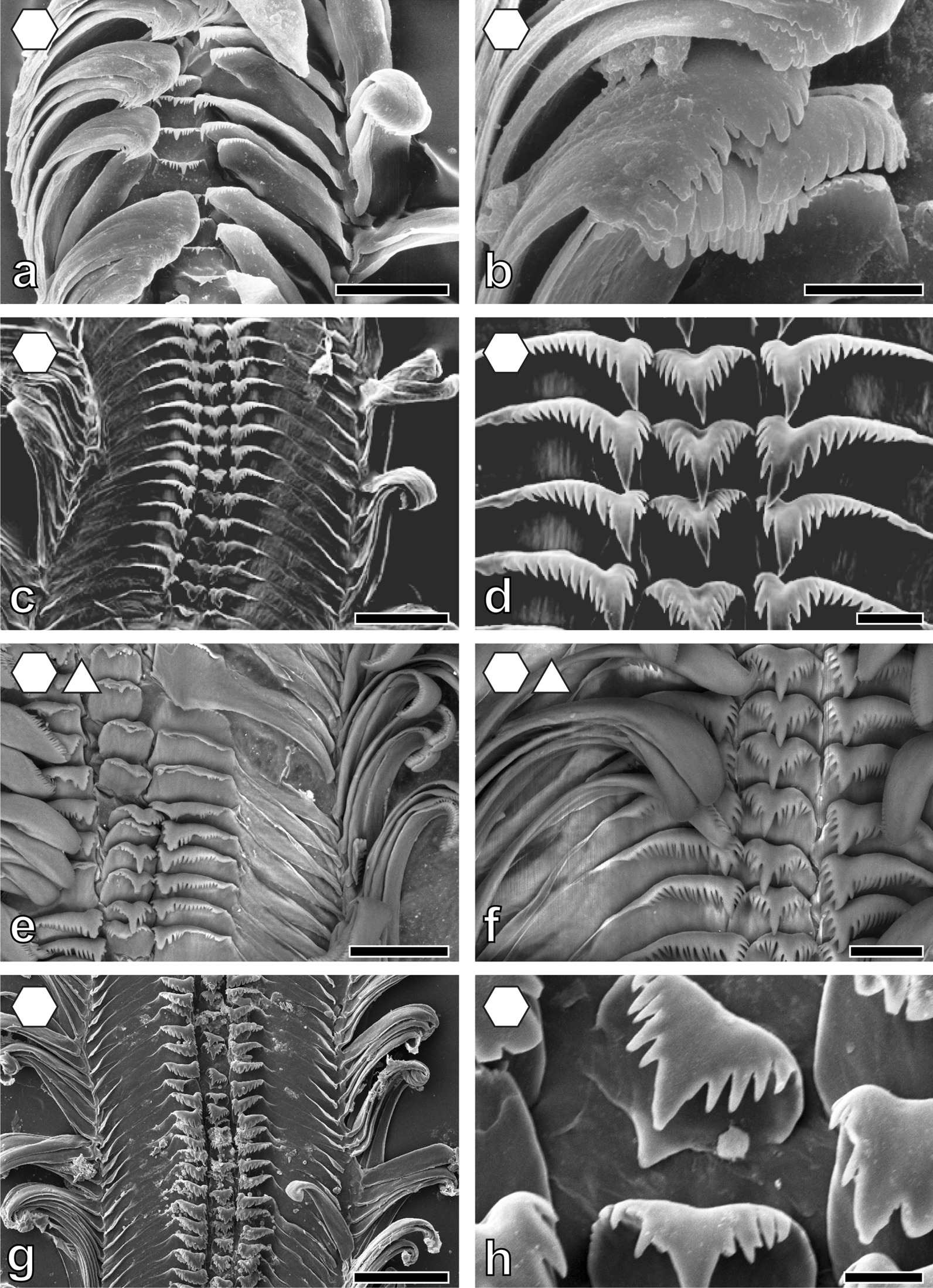
Radular teeth of: **a**, **b** *Syrnolopsis* *lacustris* ZMB 220.131, **a** overview, **b** marginals; **c**, **d** *Chytra* *kirki* IRSNB no. 63, **c** overview, **d** centrals and laterals; **e**, **f** *Limnotrochus* *thomsoni* ZMB 107.102, **e** overview, **f** centrals and laterals; **g**, **h** *Paramelania* *iridescens* ZMB 220.053, **g** overview, **h** centrals. Scale bars: **a** = 30 μm; **b**, **h** = 10 μm; **c** = 100 μm; **d**, **f** = 20 μm; **e** = 50 μm; **g** = 120 μm. Forms indicate preferred feeding substrate (circle = mud, hexagon = sand, square = plants, triangle = rock)

**Fig. 7:**
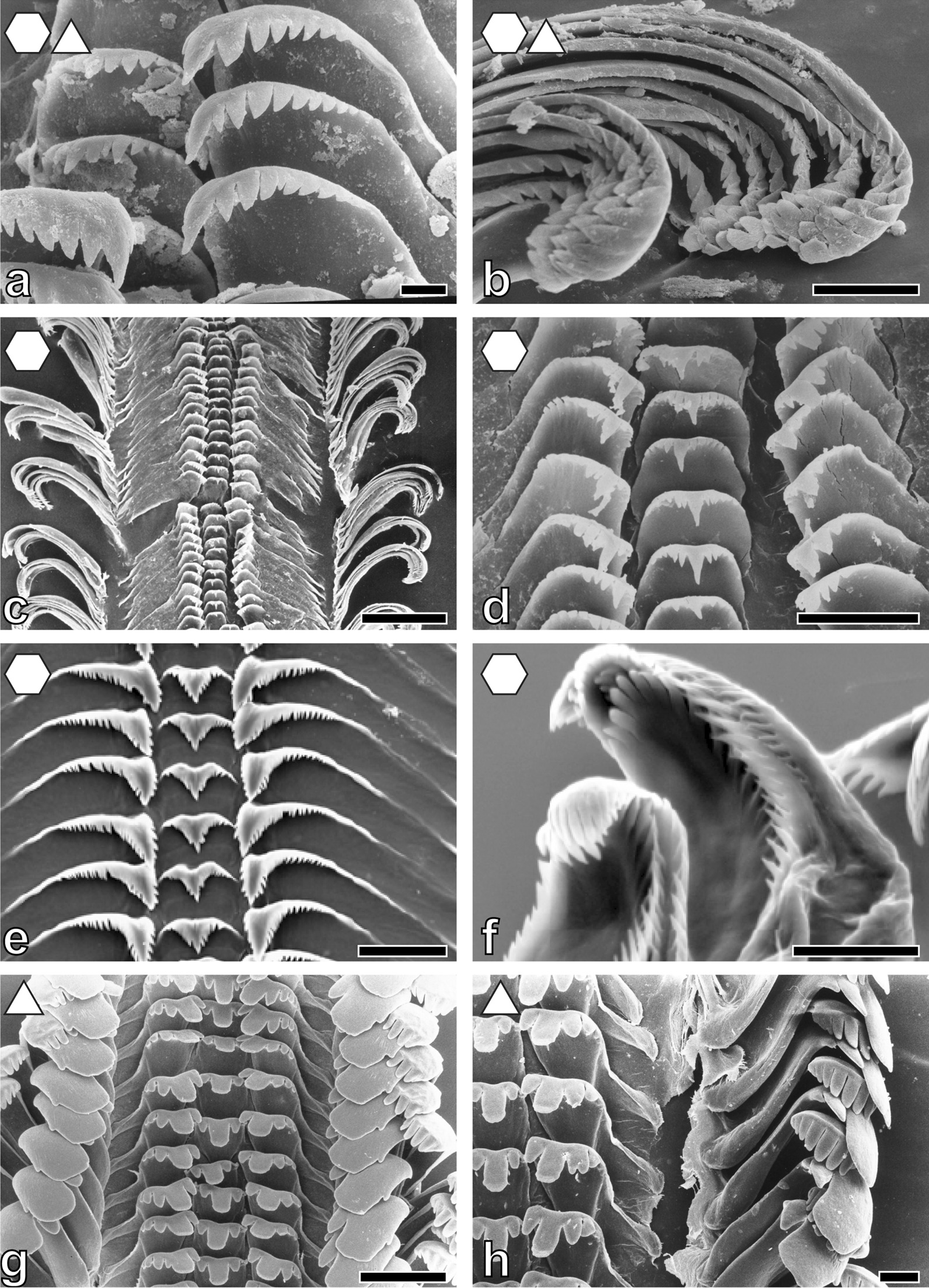
Radular teeth of: **a**, **b** *Paramelania* *damoni* ZMH without number, **a** centrals and laterals, **b** marginals; **c**, **d** *P. crassigranulata* ZMB 220.037-1, **c** overview, **d** centrals and laterals; **e**, **f** *Mysorelloides multisulcata* IRSNB no. 126, **e** centrals and laterals, **f** marginals; **g**, **h** *Lavigeria spinulosa* ZMB 220.051, **g** overview, **h** marginals. Scale bars: **a,** **f** = 10 μm; **b**, **d**, **h** = 30 μm; **c**, **g** = 100 μm; **e** = 20 μm. Forms indicate preferred feeding substrate (circle = mud, hexagon = sand, square = plants, triangle = rock)

**Fig. 8:**
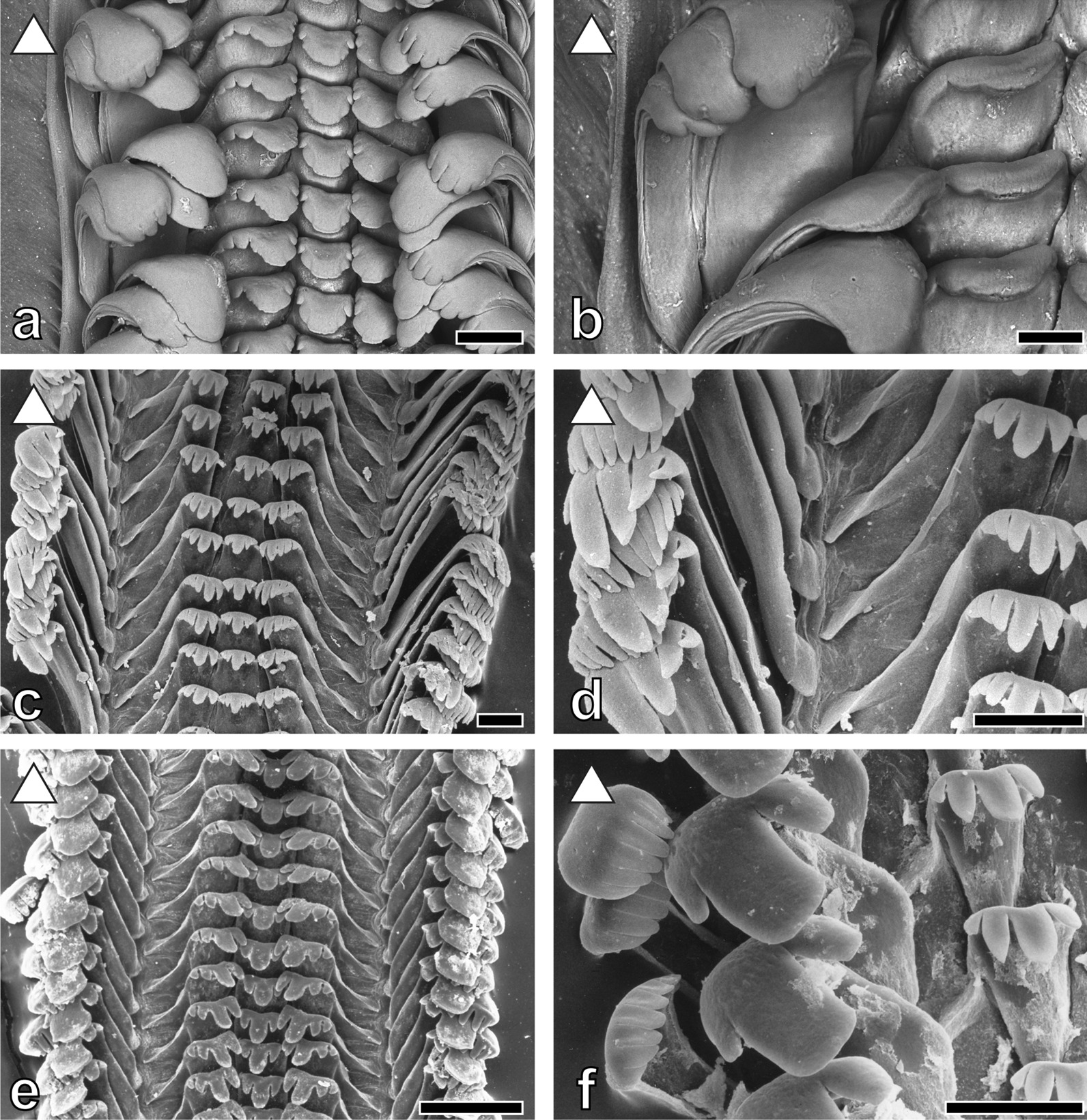
Radular teeth of: **a**, **b** *Lavigeria* *grandis* ZMH 154657/999, **a** overview, **b** laterals and marginals; **c**, **d** *L. nassa* ZMB 220.074, **c** overview, **d** laterals and marginals; **e**, **f** *L. livingstoniana* ZMB 220.117-1, **e** overview, **f** marginals and laterals. Scale bars: **a** = 100 μm; **b** = 50 μm; **c**, **d**, **f** = 30 μm; **e** = 100 μm. Forms indicate preferred feeding substrate (circle = mud, hexagon = sand, square = plants, triangle = rock)

Tooth’s morphologies (Figs. 3, 4, 5, 6, 7 and 8) can be correlated with the substrate-preference. Grazing on stones usually correlates with certain morphologies of the central tooth, either involving a spatulate, prominent central denticle (Nassopsini and *Reymondia*; Figs. 4, 8), or few or no denticles (*Bridouxia ponsonbyi*, *B. rotundata*, *B. praeclara*, *Spekia*, and *Leloupiella*; Figs. 3, 4), and with laterals bearing a prominent denticle (Nassopsini, *Reymondia*, *Bridouxia*; Figs. 3, 4, 8). Few solid-substrate feeders (*Stanleya* and *Tanganyicia*; Fig. 5) display central and lateral teeth with long denticles of the same size. Here, teeth are rather similar in their morphology to teeth of gastropods foraging on sand, possessing central, lateral, and marginal teeth with small or finger-like denticles at each cusp (Figs. 4, 5, 6, 7 and 8). All mixed substrate feeders, *Paramelania damoni*, *Limnotrochus thomsoni*, and *Bridouxia grandidieriana*, display small, finger-like denticles as well (Figs. 3, 6, 7).

**Fig. 9:**
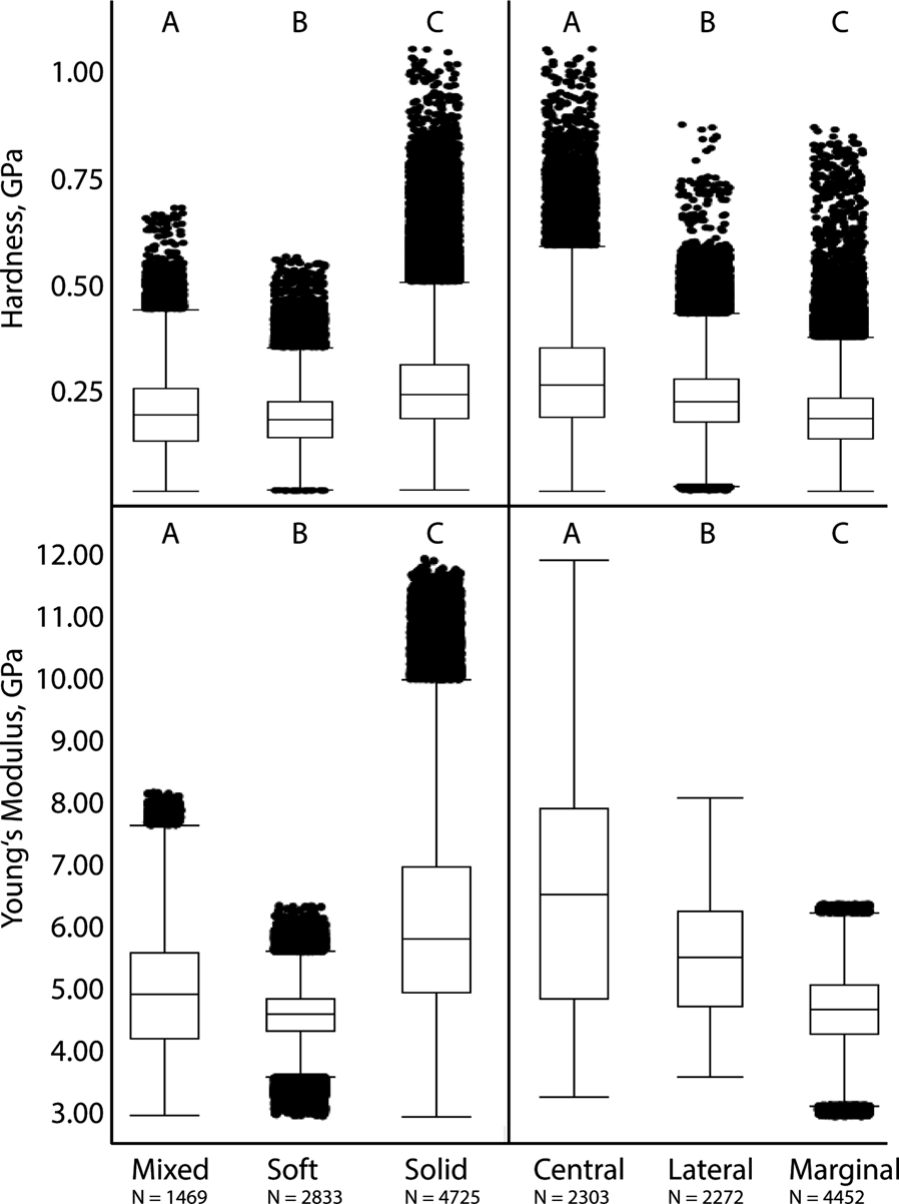
Results of nanoindentation, hardness (GPa) and Young’s modulus (GPa). (left) Comparing all tooth cusps of species feeding on mixed, soft, and solid substrate; (right) comparing all central to all lateral and all marginal tooth cups. Letters are connecting letters from Tukey–Kramer test. N = quantity of tested tooth cusps

Nanoindentation experiments provided the Young's modulus (E) as a measure of the stiffness of a solid material, describing the relationship between mechanical stress and indentation depth, and the hardness (H), the measure of the resistance to local plastic deformation. Statistical analysis of these parameters revealed normal distribution for both. Significant differences between all tooth cusps (Fig. 9) of the separate substrate feeder groups (solid, soft, and mixed) regarding both E and H were detected (p < 0.0001, F-ratio: 2, df: 59,578.92 for E, df: 20,833.04 for H). Paludomids feeding on sand have comparatively soft and flexible tooth cusps (mean ± std. deviation; E = 4.57 ± 0.45 GPa, H = 0.18 ± 0.07 GPa), species foraging on stone have the stiffest and hardest tooth cusps (E = 6.08 ± 1.52 GPa, H = 0.26 ± 0.11 GPa), and mixed substrate feeders are intermediate (E = 4.94 ± 0.99 GPa, H = 0.20 ± 0.09 GPa).

Significant differences were found between all central, lateral, and marginal tooth cusps (Fig. 9) (p < 0.0001, F-ratio: 2, df: 70,177.01 for E, df: 24,978.35 for H). Marginal teeth are comparable soft and flexible (E = 4.68 ± 0.63 GPa, H = 0.19 ± 0.08 GPa), the central teeth are comparatively hard and stiff (E = 6.48 ± 1.84 GPa, H = 0.28 ± 0.13 GPa), and the lateral ones are intermediate (E = 5.54 ± 0.92 GPa, H = 0.24 ± 0.09 GPa).

**Fig. 10:**
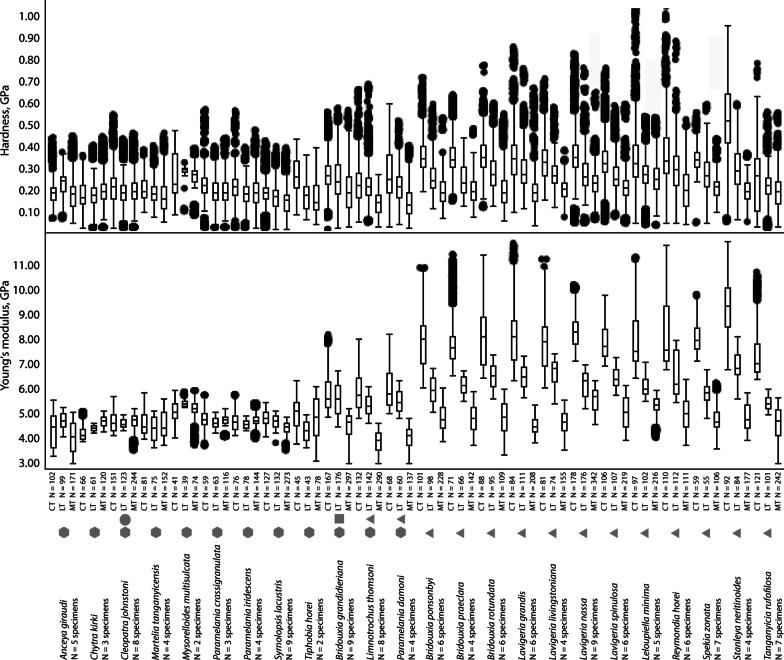
Results of nanoindentation. Hardness (GPa) and Young’s modulus (GPa) for all cusps (central, lateral, marginal, with N = quantity of measured cusps) for each species (N = quantity of measured specimens) correlated with the preferred feeding substrate (circle = mud, hexagon = sand, square = plants, triangle = rock). Connecting letters from Tukey–Kramer test can be found in Table 1

**Fig. 11:**
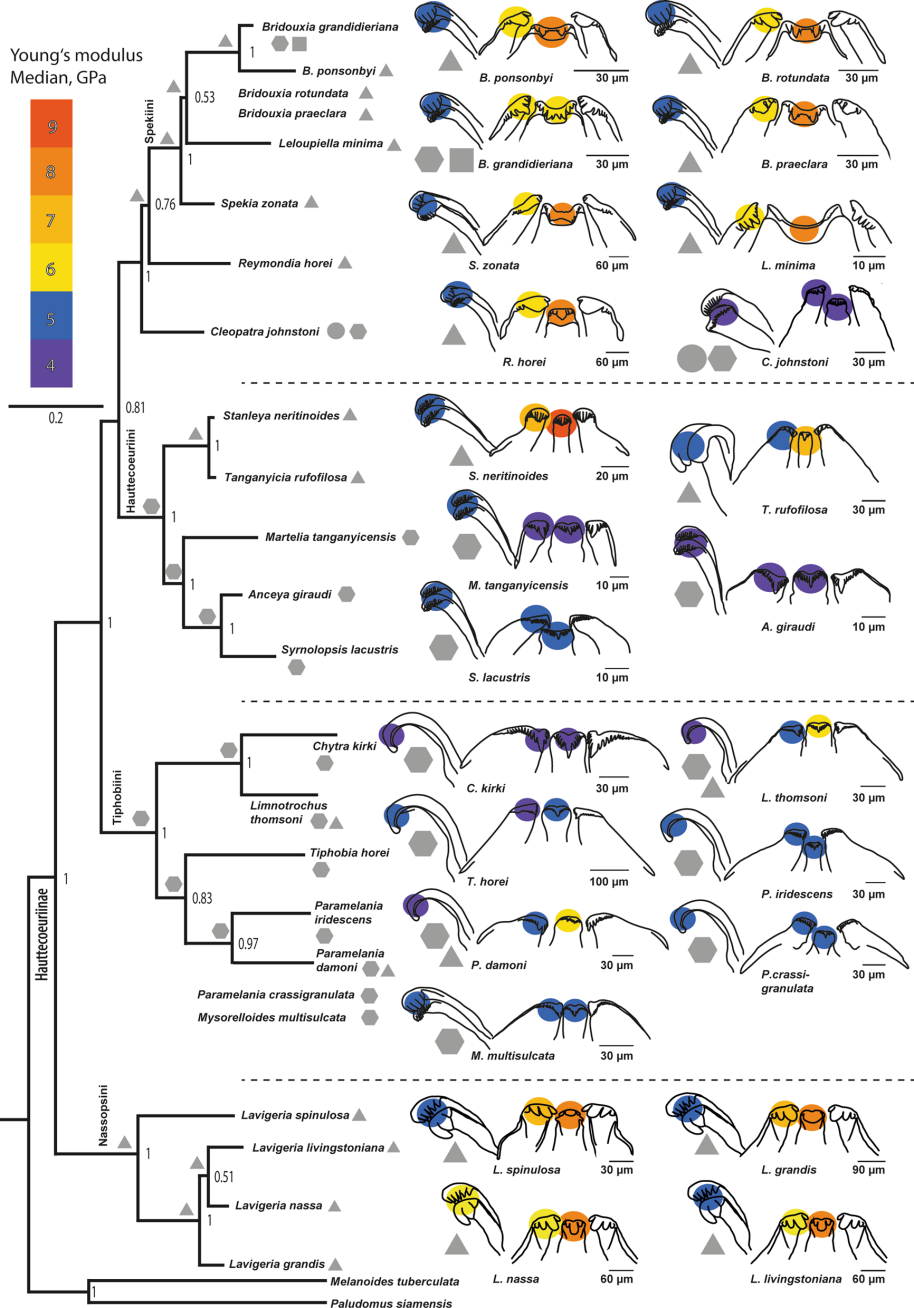
Results of nanoindentation. Median of Young’s modulus (GPa) of the central, lateral, and marginal tooth cusps for each species correlated with the preferred feeding substrate (circle = mud, hexagon = sand, square = plants, triangle = rock) against the background of a phylogenetic tree (Bayesian). Taxa without molecular information were allocated to groups based on morphological analyses from relevant literature. Reconstructed ancestral feeding substrate of the lower taxonomic levels is plotted next to the nodes

Comparing the mechanical properties within each species we consistently detect significant differences (p < 0.0001, F-ratio: 2) between central, lateral, and marginal tooth cusps (see Table 1 for all E and H values, df, and connecting letters from Tukey–Kramer test). All species feeding on solid substrate clearly display gradients in their radular properties, the stiffest and hardest parts are always the central tooth cusps, followed by the lateral ones; the softest and most flexible parts are the marginal cusps (see additionally Figs. 10, 11). In the mixed substrate feeders there’s a similar situation, central cusps are hard and stiff, lateral ones intermediate, and marginals soft and flexible; but central and lateral tooth cusps are not as distinct as in the solid substrate feeders. Species foraging on sand have quite similar mechanical properties in their tooth cusps and are more homogenous (Table 1, Figs. 10, 11).

**Table 1:** Preferred feeding substrate, Mean ± Std. Dev. for Young's modulus E (GPa) and hardness H (GPa) for each species and tooth type

Species and structure		Modulus, GPa Mean ± Std*.* Dev	Hardness, GPa Mean ± Std*.* Dev	(A) Connecting letters for comparison of E within species	(A) Connecting letters for comparison of H within species	(B) Connecting letters for comparison of E between all species and tooth types p < 0.0001 F-ratio: 71 df: 20,217.82	(B) Connecting letters for comparison of H between all species and tooth types p < 0.0001 F-ratio: 71 df: 2734.555	Feeding substrate	Species and structure		Modulus, GPa Mean ± Std*.* Dev	Hardness, GPa Mean ± Std*.* Dev	(A) Connecting letters for comparison of E within species	(A) Connecting letters for comparison of H within species	(B) Connecting letters for comparison of E between all species and tooth types p < 0.0001 F-ratio: 71 df: 20,217.82	(B) Connecting letters for comparison of H between all species and tooth types p < 0.0001 F-ratio: 71 df: 2734.555	Feeding substrate
*Bridouxia grandidieriana* N = 9 specimens				p < 0.0001 F-ratio: 2 df: 9409.887	p < 0.0001 F-ratio: 2 df: 1623.203			Mixed	*Bridouxia ponsonbyi* N = 6 specimens				p < 0.0001 F-ratio: 2 df: 24,848.35	p < 0.0001 F-ratio: 2 df: 6166.873			Solid
	CT Cusp N = 167	5*.*81 ± 0*.*81	0*.*27 ± 0*.*08	A	A	S	JK			CT Cusp N = 101	7.81 ± 0.99	0*.*35 ± 0*.*08	A	A	G	CDE	
	LT Cusp N = 176	5.55 ± 0.66	0*.*25 ± 0*.*10	B	B	T	QR			LT Cusp N = 98	5.93 ± 0.52	0*.*26 ± 0*.*08	B	B	Q	NOP	
	MT Cusp N = 297	4.47 ± 0.51	0*.*19 ± 0*.*08	C	C	M1	D1E1			MT Cusp N = 228	4.81 ± 0.56	0.19 ± 0*.*07	C	C	A1B1C1	B1C1D1	
*Limnotrochus thomsoni* N = 8 specimens				p < 0.0001 F-ratio: 2 df: 25,875.14	p < 0.0001 F-ratio: 2 df: 3296.830			Mixed	*Bridouxia praeclara* N = 4 specimens				p < 0.0001 F-ratio: 2 df: 20,873.79	p < 0.0001 F-ratio: 2 df: 4929.307			Solid
	CT Cusp N = 132	5.87 ± 0.80	0*.*23 ± 0*.*09	A	A	R	T			CT Cusp N = 71	7.92 ± 1.03	0.35 ± 0*.*08	A	A	F	CDEF	
	LT Cusp N = 142	5.31 ± 0.42	0*.*21 ± 0*.*08	B	B	X	VWX			LT Cusp N = 66	6.11 ± 0.35	0.24 ± 0*.*07	B	B	O	RS	
	MT Cusp N = 290	3.83 ± 0.42	0*.*14 ± 0*.*05	C	C	T1	M1			MT Cusp N = 142	4*.*77 ± 0.55	0.20 ± 0*.*06	C	C	C1D1E1	ZA1B1C1	
*Paramelania damoni* N = 4 specimens				p < 0.0001 F-ratio: 2 df: 11,870.71	p < 0.0001 F-ratio: 2 df: 1684.680			Mixed	*Bridouxia rotundata* N = 6 specimens				p < 0.0001 F-ratio: 2 df: 20,354.73	p < 0.0001 F-ratio: 2 df: 4417.627			Solid
	CT Cusp N = 68	6.00 ± 0.81	0*.*26 ± 0*.*12	A	A	P	MN			CT Cusp N = 88	8*.*04 ± 1*.*14	0.36 ± 0*.*09	A	A	DE	BC	
	LT Cusp N = 60	5.48 ± 0.45	0*.*21 ± 0*.*08	B	B	UV	VWX			LT Cusp N = 95	6*.*50 ± 0*.*52	0.28 ± 0*.*09	B	B	L	J	
	MT Cusp N = 137	4.03 ± 0.42	0*.*14 ± 0*.*07	C	C	S1	M1			MT Cusp N = 109	4*.*78 ± 0*.*70	0.20 ± 0*.*08	C	C	A1B1C1D1	A1B1C1	
*Anceya giraudi* N = 5 specimens				p < 0.0001 F-ratio: 2 df: 1598.901	p < 0.0001 F-ratio: 2 df: 560.1817			Soft	*Lavigeria grandis* N = 6 specimens				p < 0.0001 F-ratio: 2 df: 38,217.95	p < 0.0001 F-ratio: 2 df: 3899.479			Solid
	CT Cusp N = 102	4.30 ± 0.69	0*.*19 ± 0*.*05	A	A	P1Q1	E1F1			CT Cusp N = 84	8.07 ± 1.11	0.34 ± 0*.*13	A	A	D	EF	
	LT Cusp N = 99	4.67 ± 0.32	0*.*22 ± 0*.*05	B	B	F1	TU			LT Cusp N = 111	6.45 ± 0.46	0.29 ± 0*.*09	B	B	LM	I	
	MT Cusp N = 171	4*.*03 ± 0.59	0*.*18 ± 0*.*07	C	C	S1	G1H1I1			MT Cusp N = 208	4.50 ± 0.36	0.19 ± 0*.*08	C	C	L1M1	D1E1F1	
*Chytra kirki* N = 3 specimens				p < 0.0001 F-ratio: 2 df: 8124.837	p < 0.0001 F-ratio: 2 df: 157.4867			Soft	*Lavigeria livingstoniana* N = 4 specimens				p < 0.0001 F-ratio: 2 df: 20,914.61	p < 0.0001 F-ratio: 2 df: 3411.923			Solid
	CT Cusp N = 66	4*.*17 ± 0.26	0*.*17 ± 0*.*06	C	C	R1	J1K1			CT Cusp N = 81	7.74 ± 0.97	0.33 ± 0*.*09	A	A	H	H	
	LT Cusp N = 61	4*.*38 ± 0.12	0*.*17 ± 0*.*06	B	B	N1O1	I1J1			LT Cusp N = 74	6.69 ± 0.45	0*.*27 ± 0*.*07	B	B	K	JK	
	MT Cusp N = 120	4*.*66 ± 0.21	0*.*19 ± 0*.*07	A	A	F1G1	E1F1			MT Cusp N = 155	4.62 ± 0.48	0*.*20 ± 0*.*04	C	C	G1H1	ZA1B1	
*Cleopatra johnstoni* N = 8 specimens				p < 0.0001 F-ratio: 2 df: 252.9942	p < 0.0001 F-ratio: 2 df: 205.9801			Soft	*Lavigeria nassa* N = 9 specimens				p < 0.0001 F-ratio: 2 df: 44,599.76	p < 0.0001 F-ratio: 2 df: 4134.603			Solid
	CT Cusp N = 151	4*.*65 ± 0*.*42	0*.*21 ± 0*.*08	B	A	F1G1H1	VWXY			CT Cusp N = 178	8.40 ± 0.85	0*.*36 ± 0*.*11	A	A	B	B	
	LT Cusp N = 123	4.56 ± 0.18	0*.*18 ± 0*.*06	C	C	I1K1	F1G1H1			LT Cusp N = 176	6.19 ± 0.53	0*.*27 ± 0*.*08	B	B	N	KL	
	MT Cusp N = 244	4.68 ± 0.31	0*.*19 ± 0*.*06	A	B	F1	D1E1F1			MT Cusp N = 342	5.53 ± 0.50	0*.*24 ± 0*.*07	C	C	TU	S	
*Martelia tanganyicensis* N = 4 specimens				p < 0.0001 F-ratio: 2 df: 116.8776	p < 0.0001 F-ratio: 2 df: 244.2914			Soft	*Lavigeria spinulosa* N = 6 specimens				p < 0.0001 F-ratio: 2 df: 29,162.21	p < 0.0001 F-ratio: 2 df: 3134.948			Solid
	CT Cusp N = 81	4*.*55 ± 0.49	0*.*20 ± 0*.*05	A	A	I1J1K1L1	XYZA1			CT Cusp N = 106	7.88 ± 0.68	0*.*33 ± 0*.*10	A	A	F	GH	
	LT Cusp N = 75	4.35 ± 0.48	0*.*18 ± 0*.*05	B	B	O1P1	E1F1G1H1			LT Cusp N = 107	6.42 ± 0.40	0*.*26 ± 0*.*08	B	B	M	MN	
	MT Cusp N = 152	4.52 ± 0.53	0*.*17 ± 0*.*07	A	C	K1L1	J1K1			MT Cusp N = 219	5.01 ± 0.63	0*.*21 ± 0*.*07	C	C	Z	WXY	
*Mysorelloides multisulcata N = 2 specimens*				p < 0.0001 F-ratio: 2 df: 337.2236	p < 0.0001 F-ratio: 2 df: 109.8094			Soft	*Leloupiella* *minima* N = 5 specimens				p < 0.0001 F-ratio: 2 df: 15,762.00	p < 0.0001 F-ratio: 2 df: 739.7396			Solid
	CT Cusp N = 41	5.06 ± 0.43	0*.*26 ± 0*.*10	C	B	YZ	LMNO			CT Cusp N = 92	7.99 ± 1.22	0*.*34 ± 0*.*16	A	A	E	F	
	LT Cusp N = 39	5.39 ± 0.13	0*.*28 ± 0*.*03	A	A	VW	IJ			LT Cusp N = 84	6.06 ± 0.39	0*.*27 ± 0*.*08	B	B	OP	KLM	
	MT Cusp N = 74	5.12 ± 0.35	0*.*25 ± 0*.*06	B	C	Y	PQR			MT Cusp N = 177	5.30 ± 0.37	0*.*25 ± 0*.*09	C	C	X	OPQ	
*Paramelania crassigranulata* N = 3 specimens				p < 0.0001 F-ratio: 2 df: 212.7667	p < 0.0001 F-ratio: 2 df: 221.3736			Soft	*Reymondia horei* N = 6 specimens				p < 0.0001 F-ratio: 2 df: 17,244.19	p < 0.0001 F-ratio: 2 df: 2694.074			Solid
	CT Cusp N = 59	4.76 ± 0.36	0*.*22 ± 0*.*08	A	A	B1C1D1E1	TU			CT Cusp N = 97	8.21 ± 1.34	0*.*35 ± 0*.*15	A	A	C	BCD	
	LT Cusp N = 63	4.61 ± 0.21	0*.*19 ± 0*.*07	C	B	F1G1	C1D1E1F1			LT Cusp N = 102	6.48 ± 0.85	0*.*29 ± 0*.*11	B	B	L	I	
	MT Cusp N = 116	4.67 ± 0.24	0*.*19 ± 0*.*07	B	B	G1H1I1J1	D1E1F1			MT Cusp N = 216	4.99 ± 0.65	0*.*20 ± 0*.*09	C	C	Z	YZ	
*Paramelania iridescence* N = 4 specimens				p < 0.0001 F-ratio: 2 df: 401.3311	p < 0.0001 F-ratio: 2 df: 218.9492			Soft	*Spekia zonata* N = 7 specimens				p < 0.0001 F-ratio: 2 df: 11,499.22	p < 0.0001 F-ratio: 2 df: 1089.918			Solid
	CT Cusp N = 76	4.65 ± 0.38	0*.*22 ± 0*.*08	B	A	F1G1H1	UV			CT Cusp N = 110	8*.*09 ± 0*.*65	0*.*34 ± 0*.*07	A	A	D	DEFG	
	LT Cusp N = 78	4.55 ± 0.18	0*.*18 ± 0*.*06	C	B	K1L1	E1F1G1H1			LT Cusp N = 112	5.78 ± 0.42	0.27 ± 0*.*09	B	B	S	JKLM	
	MT Cusp N = 144	4.73 ± 0.24	0*.*18 ± 0*.*07	A	B	E1	E1F1G1			MT Cusp N = 111	4.74 ± 0.50	0.21 ± 0*.*07	C	C	D1E1	VW	
*Syrnolopsis lacustris* N = 9 specimens				p < 0.0001 F-ratio: 2 df: 5510.110	p < 0.0001 F-ratio: 2 df: 842.0682			Soft	*Stanleya neritinoides* N = 4 specimens				p < 0.0001 F-ratio: 2 df: 26,627.28	p < 0.0001 F-ratio: 2 df: 5043.367			Solid
	CT Cusp N = 127	4.82 ± 0.29	0*.*18 ± 0*.*06	A	A	A1	G1H1I1			CT Cusp N = 59	9.32 ± 1.09	0.51 ± 0*.*18	A	A	A	A	
	LT Cusp N = 132	4.64 ± 0.26	0*.*16 ± 0*.*06	B	B	F1G1H1	L1			LT Cusp N = 55	6.96 ± 0.55	0.29 ± 0*.*12	B	B	J	I	
	MT Cusp N = 273	4.41 ± 0.21	0*.*14 ± 0*.*06	C	C	N1	M1			MT Cusp N = 106	4.83 ± 0.53	0.19 ± 0*.*06	C	C	A1B1	D1E1F1	
*Tiphobia horei* N = 2 specimens				p < 0.0001 F-ratio: 2 df: 298.3816	p < 0.0001 F-ratio: 2 df: 695.6014			Soft	*Tanganyicia rufofilosa* N = 7 specimens				p < 0.0001 F-ratio: 2 df: 24,804.23	p < 0.0001 F-ratio: 2 df: 420.1531			Solid
	CT Cusp N = 45	4.97 ± 0.65	0*.*26 ± 0*.*08	A	A	Z	KLMN			CT Cusp N = 121	7.27 ± 0.80	0.24 ± 0*.*14	A	A	I	R	
	LT Cusp N = 43	4.26 ± 0.42	0*.*17 ± 0*.*07	C	B	Q1	H1I1J1K1			LT Cusp N = 101	5*.*40 ± 0.29	0*.*21 ± 0*.*07	B	B	W	VWX	
	MT Cusp N = 78	4.78 ± 0.86	0*.*16 ± 0*.*08	B	C	A1B1C1D1E1	K1L1			MT Cusp N = 242	4*.*61 ± 0.60	0*.*19 ± 0*.*08	C	C	H1J1	D1E1	

Connecting letters from Tukey–Kramer test (which are usually plotted above the boxplots in Fig. 10) are listed under (A) and (B) identifying homogenous groups for (A) comparing the tooth types [CT, LT, and MT] within a single species and (B) comparing all species and structures

*CT* central tooth, *LT* lateral tooth, *MT* marginal tooth

When comparing E and H of each tooth type between all species significant differences were detected (p < 0.0001, F-ratio: 71, df: 20,217.82 for E, df: 2734.555 for H; see Figs. 10, 11; see Table 1, columns B for connecting letters from Tukey–Kramer test).

Two-way ANOVA (see Additional file 1) revealed that feeding substrate and tooth type both have same significant effect on E and H values (p < 0.0001 for each, see Additional file 1: Tables S2 and S3 for df, F-ratio, interaction terms). Least square mean plots (Additional file 1: Figure S1) reveal that E and H values of the central teeth are more influenced by the feeding substrate that the lateral teeth, and finally marginal teeth.

**Fig. 12:**
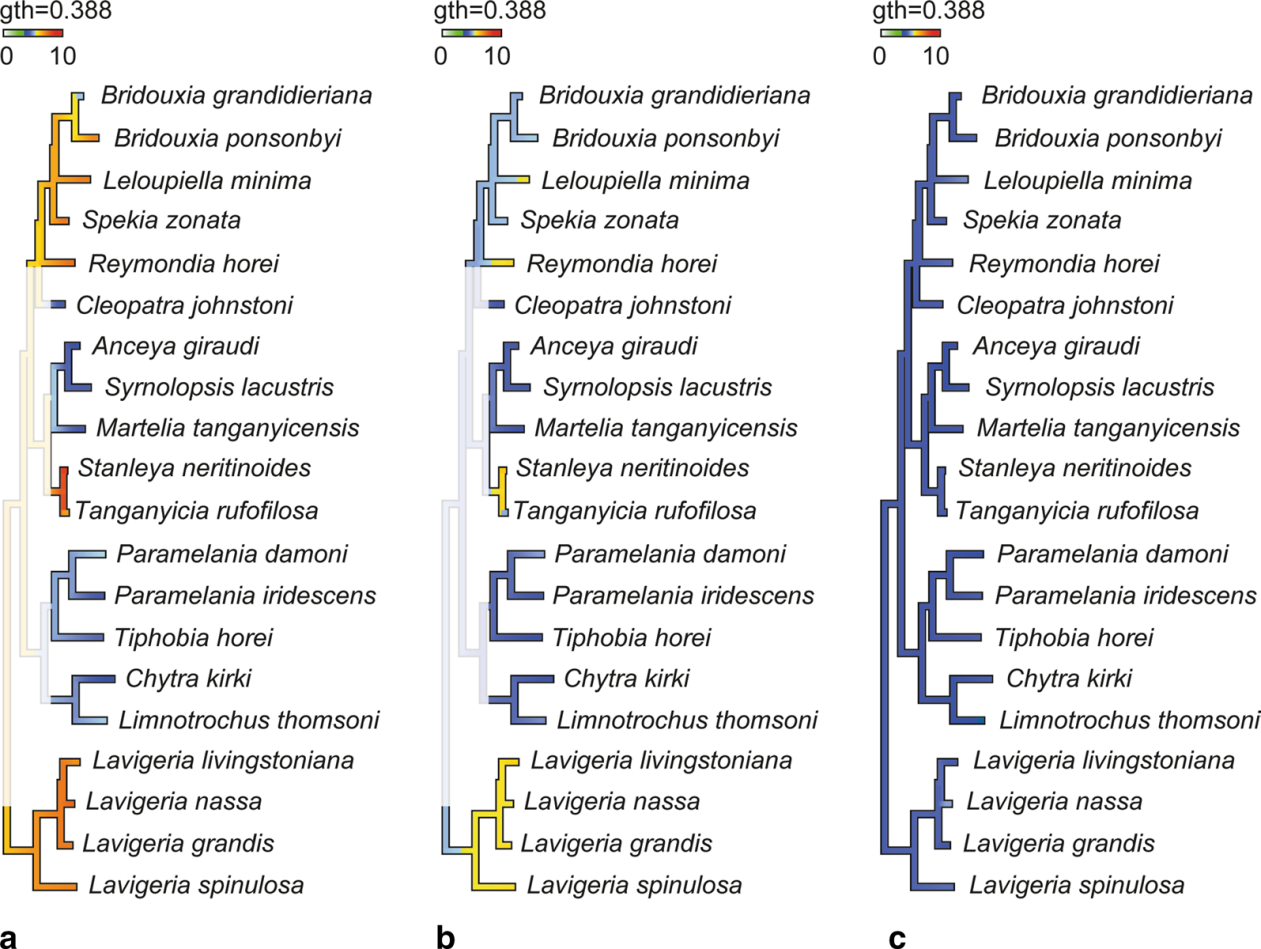
Changes of Young’s modulus over the phylogeny (excl. outgroups) visualized using continuous character mapping (from 5 GPa [blue] to 10 GPa [red]) for **a** central tooth, **b** lateral tooth, **c** marginal tooth for the lower taxonomic levels

Reconstruction of the ancestral feeding substrate (Fig. 11) suggests a solid substrate for the Spekiini and *Reymondia* accompanied by central teeth of 7–8 GPa Young’s modulus and lateral teeth of 6 GPa, which convergently increased stiffness to 8 GPa in *Leloupiella* and *Reymondia* (Fig. 12). Within the Spekiini *Bridouxia grandidieriana* adapted to feeding on mixed substrate by reducing the Young’s modulus in the central teeth to 6 GPa (Fig. 12). A soft ancestral feeding substrate was computed for the Hauttecoeuriini (Fig. 11), here accompanied by a subsequent shift to solid substrate in the group containing *Stanleya* and *Tanganyicia* with an increasing Young’s modulus in both central and lateral teeth, and for the Tiphobiini, followed by a parallel adaptation to mixed feeding substrate in *Paramelania damoni* and *Limnotrochus thomsoni* in connection with an increase of the Young’s modulus from 5 to 6 GPa in the central teeth (Fig. 12). For the Nassopsini a solid feeding substrate was reconstructed (Fig. 11), here the Young’s modulus of all tooth types remains similar. For the marginal teeth no changes in the Young’s modulus were detected since all analysed species have similar mechanical properties in this tooth type (Fig. 12).

## Discussion

The gastropods in Lake Tanganyika have limited options regarding their habitat, as they occur below the surf zone down to 200 m, with the deeper parts of the lake containing little oxygen and are toxic [44]. Sharing habitats might result in considerable inter- and intraspecific competition, but we found strong evidences for the avoidance or the reduction of resource competition by clear substrate-specificity in most paludomid groups [see also 44]. In Group 1, Spekiini Ancey, 1906 [58], *Reymondia* Bourguignat, 1885 [45] and riverine Cleopatrini Pilsbry and Bequaert, 1927 [60], Group 2, Hauttecoeuriini Bourguignat, 1885 [45], Syrnolopsini, Bourguignat, 1890 [47], and Group 3, Tiphobiini Bourguignat, 1886 [46], (groups in accordance with [42, 44]) some species feed on biofilm that covers stones (solid substrate), some select algae from sandy and muddy surfaces (soft substrate) and few (mixed) feed either on both (*Paramelania damoni*, *Limnotrochus thomsoni*) or on algae attached to plants and covering sand (*Bridouxia grandidieriana*). Group 4, containing *Lavigeria* and riverine *Potadomoides*, consist probably of species that exclusively feed on solid substrate. Unfortunately, reliable data on preferred substrate is not available for *Potadomoides* which has not been found again in the last decades. Its localities, the Malagarasi River and the Congo River drainage, are characterized by swampy areas as well as rapids with rocks. But, since its radular tooth characters are similar to *Lavigeria* species [43], we rather conclude that *Potadomoides* also feeds on algae from solid substrates.

The mechanical properties (E, H) of the paludomid radular teeth correlate with the preferred substrate and reflect different eco-morphotypes (Fig. 11). All species foraging on stones, viz. *Bridouxia ponsonbyi*, *B. rotundata*, *B. praeclara*, *Leloupiella minima*, *Spekia zonata*, *Reymondia horei*, *Stanleya neritinoides*, *Tanganyicia rufofilosa*, *Lavigeria spinulosa*, *L. livingstoniana*, *L. nassa*, and *L. grandis*, show gradual and distinct differences in their stiffness and hardness between the tooth types, which can be explained by different functional loads (Figs. 10, 11). The material properties certainly influence the mechanics of structures, the Young’s modulus E is, for example, directly linked with the ability of a structure to transfer forces [e.g. 131, 132, 133, 134] and correlates with the structures mechanical behaviour while puncturing and in direct turn the resistance of structures to failure [e.g. 135, 136]. We anticipate here that the stiff central and lateral teeth are rather used for scratching across the solid feeding substrate removing food items attached to it [see also 121, 129]; this function of the CT was also documented for *Dendronotus* [see 119], transferring higher force from the radular muscles via the tooth cusps onto the ingesta. The softer and more flexible marginal teeth have a smaller ability to transfer forces necessary to loosen a tightly attached biofilm. But their elasticity allows the reduction of the stress concentration, for example in case of hitting the substrate asperities. Their mechanical properties enable them to catapult back to place, possibly without fractures or ruptures, after hitting an obstacle. We would hence deduce that the marginal teeth are rather functionally different from the central and lateral teeth, possibly harvesting, like a broom, food items or particles that had been loosened from the substrate by grinding action of the central and lateral teeth [see also 108, 121, 129, 137]. This type of radula is considered to be a multifunctional tool.

For all species foraging on sand or mud, viz. *Cleopatra johnstoni*, *Martelia tanganyicensis*, *Anceya giraudi*, *Syrnolopsis lacustris*, *Chytra kirki*, *Tiphobia horei*, *Paramelania iridescens*, *P. crassigranulata*, and *Mysorelloides multisulcata*, we found similar mechanical properties in all tooth types as well as comparably soft and flexible tooth cusps (Figs. 10, 11). Their hardness and elasticity values are comparable to the mechanical properties of the solid substrate feeders’ marginal teeth. We would hence propose that these species rather possess a monofunctional radula with each tooth serving as broom collecting algae from the surface. The mechanical properties of the mixed substrate feeders, viz. *Bridouxia grandidieriana*, *Limnotrochus thomsoni*, and *Paramelania damoni*, are intermediate, as they have slightly softer and more flexible central and lateral tooth cusps compared to the gastropods loosening algae from stones but stiffer and harder ones than the species selecting biofilm from sand (Figs. 10, 11). Even though the gradients between the tooth cusps are not as distinct as in the gastropods foraging on stone, the existence of the gradual differences leads to the conclusion that, despite of softer and more flexible tooth cusps, the mixed substrate feeders also possess a multifunctional radula. Central and lateral teeth are rather used for loosening food items, whereas the softer marginal tooth cusps, showing similar properties to the marginal teeth of solid feeders and to each tooth type of species selecting algae from sand, serve as brooms.

Past studies on Sacoglossa revealed that tooth morphologies between closer related taxa differ because of specialisation to distinct ingesta [113–116] whereas for *Dendronotus* (Nudibranchia) it was reported that morphology relates to both phylogeny and ecology [119]. For paludomids we found that tooth’s morphologies correlate with the preferred feeding substrate. In most solid substrate feeders central teeth displaying either a prominent denticle (Nassopsini and *Reymondia*; Figs. 4, 8), or few or no denticles (*Bridouxia ponsonbyi*, *B. rotundata*, *B. praeclara*, *Spekia*, and *Leloupiella*; Figs. 3, 4), as well as laterals bearing a prominent denticle (Nassopsini, *Reymondia*, *Bridouxia*; Figs. 3, 4, 8), allow a large interaction surface between tooth cusps and ingesta directly transferring force. Additionally, these teeth are rather short and broad, probably leading to the reduction of deformation when tensile and compressive stresses appear in the structure during this action. However, some solid feeders (*Stanleya* and *Tanganyicia*; Fig. 5) as well as the mixed substrate feeders (*Paramelania damoni*, *Limnotrochus thomsoni*, *Bridouxia grandidieriana*; Figs. 3, 6, 7) display an alternative morphology, rather similar to teeth of gastropods foraging on sand. Soft substrate feeders possessing central, lateral, and marginal teeth with small or finger-like denticles at each cusp (Figs. 4, 5, 6 and 7), which probably enables them to rake between the grains gathering the food particles. We hypothesize that radular tooth performance in *Stanleya*, *Tanganyicia*, and all mixed substrate feeders is ensured by mechanical properties rather than morphology. Thus, pure morphology does not consistently reflect adaptations, but the morphology furnished by mechanical properties allows the establishment of tooth eco-morphotypes.

Adaptations to distinct substrates, solid as well as soft and mixed, are present in most taxonomic paludomid lineages (Fig. 11). This in turn leads to the hypothesis that one main engine of their evolution is trophic specialization to feeding substrates, establishing distinct ecological niches and allowing the coexistence of taxa [see also 119 for diet-driven radiation in *Dendronotus*]. Only Group 4 (Nassopsini Kesteven, 1903 [138], contains *Lavigeria*) is exceptional because it contains exclusively solid substrate feeders (Fig. 11). *Lavigeria* is a paludomid group containing a plethora of named species [see e.g. 79, 139] that had been treated as result of an exclusively intralacustrine adaptive radiation. Unfortunately, a systematic revision identifying evolutionary entities is still lacking, hindering hypotheses on their evolution. However, when comparing *Lavigeria* adult shells their distinct sizes are apparent which are interpreted as result of annidation through different body sizes [unpublished data]. Also, *Lavigeria* radular teeth are of distinct tooth sizes. *Lavigeria grandis* displaying the largest teeth, followed by *L. nassa*, *L. spinulosa*, and finally *L. livingstoniana* possessing the smallest teeth (Figs. 7, 8). This could be an indication that *Lavigeria* species avoid competition by trophic specialization, albeit not to different feeding substrates, but rather to different algae or biofilm types growing on solid substrates. However, in order to determine whether species have distinct food preferences, it would be necessary to collect and gather feeding substrates and biofilms directly in situ. The only available collectors’ comments on Lake Tanganyikan biofilms, however, suggest that paludomids feed on soft algae, overcasting either rocks or plant structures or covering sandy or muddy substrates. This could explain the relatively soft and elastic tooth cusp of these gastropods in comparison with published hardness and elasticity measurements on teeth of hard calcified algae feeders, such as e.g. Polyplacophora and Patellogastropoda (with E ranging from 16 GPa over 90–125 GPa up to 52–140 GPa [140–144] and H ranging from 9–12 GPa [140, 142, 144]).

Molecular clock approaches [42, 122] support an ancient origin of diversity and disparity, long before the formation of Lake Tanganyika or a proto-lake. After the rifting of the African continent and the formation of the lake several independent colonialization events of already distinct riverine paludomid lineages succeeded from the surrounding river systems. We here reconstructed ancestral feeding substrates and ancestral E values for the distinct tooth types [for ancestral state reconstruction and diet preference evolution in Nudibranchia see also 145]. However, this reconstruction is only reliable for the lower taxonomic groups (at the level of Hauttecoeuriini, Tiphobiini, Nassopsini, or the Group containing the Spekiini and *Reymondia*), but not on the level of the Hauttecoeuriinae. Due to the fact that we have tested only 24 species of the flock by nanoindentation, which is a highly laborious experimental set-up, we lack reliable information for many species (e.g. *Bathanalia*, many *Lavigeria* species). However, by including more paludomid taxa in our molecular tree and adding feeding substrate information we found evidence that the ancestral riverine feeding substrate of the Hauttecoeuriinae is of soft nature [122] which could have been accompanied by long and slender teeth with numerous denticles of equal size (monomorphic radula) and indicative of an preadaptation sensu strictu [146] to the riverine substrate. This was possibly succeeded by the convergent shift to solid substrate in two paludomid lineages (ancestor of (i) Spekiini and *Reymondia* as well as (ii) Nassopsini) evolving central and lateral tooth morphologies adapted to this substrate furnished by the evolution of harder and stiffer central and lateral tooth cusps. Since we unfortunately lack biomaterial property information as well as reliable feeding substrate information for *Potadomoides*, the riverine sister group of the Nassopsini [43], we do not know if (a) the ancestor of the Nassopsini or (b) the ancestor of the group containing the Nassopsini and *Potadomoides* has adapted to solid substrate. If *Potadomoides* species fed also on solid substrate it would be possible that a shift from soft to solid feeding substrate has taken place in riverine rapids. Additionally, possibly after the origin and the colonialization of Lake Tanganyika, the ancestor of *Stanleya* and *Tanganyicia* has adapted to solid substrate. Nevertheless, *Stanleya*, and *Tanganyicia* display (Fig. 5), as described above, rather monomorphic radular teeth similar to the soft substrate feeders. These taxa probably still carry their ancestral riverine morphological characters. Thus, their adaptation to solid substrate involved only the change in material properties hardness and elasticity.

*Bridouxia* is probably a case of an exclusively intralacustrine adaptive radiation, strongly indicated by molecular clock approaches [122]. Here we were able to detect secondary adaptation to mixed feeding substrate for *B. grandidieriana*. This taxon became probably adapted through changes in morphology as well as material properties, evolving softer and more flexible central and lateral teeth with small denticles serving as a broom (Fig. 3). The mixed substrate feeders *Paramelania damoni* and *Limnotrochus thomsoni* retained their ancestral tooth morphologies (Figs. 6, 7), but temporary solid substrate feeding is probably enabled by the evolution of stiffer and harder central and lateral tooth cusps. All other lacustrine species (*Martelia*, *Anceya*, *Syrnolopsis*, *Chytra*, *Tiphobia*, *P. iridescens*, *P. crassigranulata*, *Mysorelloides*) are adapted to foraging on soft substrates carrying their ancestral riverine tooth morphologies [122] as well as displaying soft and flexible teeth (Figs. 10, 11).

In summary, tooth shapes and tooth mechanical properties differ more than expected in sister taxa (e.g. *Bridouxia*, *Lavigeria* [here through tooth size], or between all sister groups as e.g. Tiphobiini and Hauttecoeuriini). Additionally, parallel evolution of tooth shapes and mechanical properties can be detected (*Reymondia* and Nassopsini). This suggests that radular teeth in paludomids are under strong selection and that diverging from close relatives has often been favoured resulting in the micro partitioning of the environment; this is similar to ingesta-processing structures (beaks, skull bones) found in other radiations with trophic specialisation being one main driving force (e.g. Darwin finches, cichlid fish).

## Conclusion

Here we present the first comparative study on the mechanical properties, hardness and elasticity, of taenioglossan radular teeth from African paludomid gastropods from Lake Tanganyika and surrounding river systems, based on a large sample size and in a phylogenetic and ecological context. The tested paludomid teeth correlate with their preferred feeding substrate and reflect different tooth eco-morphotypes accompanying morphology. Our identification of adaptations allows to put forward a new perspective on the evolution of this species flock. We postulate that trophic specialisation resulting in niche partitioning has played a major role in the evolution and radiation of this flock.

## Methods

As basis we used paludomid gastropods (Fig. 1) collected in earlier studies [see 44], supplemented by additional material of taxa collected by Heinz Büscher, Basel. Specimens stored in ethanol are inventoried at the Museum für Naturkunde Berlin (ZMB), the Musée royal de l’Afrique centrale, Tervuren, Belgium (MRAC), the Royal Belgian Institute of Natural Sciences, Brussels, Belgium (IRSNB), and the Zoological Museum (ZMH) of the Center of Natural History (CeNak) in Hamburg (for details on sampling locality see Additional file 1: Table S1). Specimens were identified based on shell morphology in comparison with type material following essentially [44] and literature referred to therein.

For nanoindentation [detailed descripting of method in 129, 130] overall 124 radulae belonging to 24 species (Additional file 1: Table S1), accompanied by data on 7 specimens from *Spekia zonata* taken from [129], were manually extracted from adult specimens, freed from surrounding tissues, dried and, laying on its side, tapped with double-sided adhesive tape to a glass object slide. The tape ensured that the radulae were accurately arranged, with marginal teeth at the bottom, followed by lateral, central, lateral, and on the top marginal teeth. This procedure ensured that after polishing only one tooth type was superficial at the plain surface. Each radula was surrounded by a small metallic ring resulting an almost parallel sample after polishing necessary for an almost error-free nanoindentation. Rings were filled with epoxy resin (RECKLI^®^EPOXIWST, Young’s modulus of the epoxy is 1 GPa), known to not infiltrate the teeth, polymerizing at room temperature. Object slide and tape were removed, radulae were polished with gradual diamond pastes (Buehler MetaDi Ultra Paste 6 µm 3 µm, 1 µm) and smoothened with a polishing machine (Buehler MataServ 250 with Struers OP-U, 0.04 µm suspension with 250 rpm) for a plain surface displaying the longitudinal section of teeth (Fig. 2c–f). After performing nanoindentation, employing a Nanoindenter SA2 (MTS Nano Instrument, Oak Ridge, TN, USA; CSM) equipped with Berkovich indenter tip, on the superficial tooth row (marginals) samples were again smoothened until the next tooth row (laterals) was on display (Fig. 2b). Steps were repeated until all teeth were measured. The indents for this study were made at the tooth cusps with each indentation curve controlled manually for correct surface finding. In each specimen, about 25 tooth rows of the outer wear zone were tested, resulting in more than 9027 measured tooth cusps for all analysed specimens. We focussed on this specific radular locality to exclude not matured teeth from this study. For detailed quantity [N] of specimens per species, evaluable indentation data on hardness/elasticity per species and per tooth type see Table 1 and Fig. 10 (N of analysed specimens differs between species due to availability of radular material; not every nanoindentation curve and resulting data was evaluated due to artefacts, e.g. surface finding problems, local surface roughness, the angle between the embedded tooth and the epoxy surface; thus N of analysed tooth cusps differs between specimens). Reliable nanoindentation curves and resultant data tables on Young’s modulus (Elasticity modulus; E) and hardness (H) of materials were exported; values of E and H were either determined at penetration depths of 480–520 nm (for larger teeth, in *Spekia*, *Reymondia*, *Lavigeria*, *Chytra*) or at penetration depth 450–500 nm (for smaller teeth, in *Bridouxia*, *Leloupiella*, *Cleopatra*, *Stanleya*, *Tanganyicia*, *Martelia*, *Anceya*, *Syrnolopsis*, *Limnotrochus*, *Paramelania*, *Mysorelloides*) with about 30 values per indentation. These indentation depths were targeted, because at low depths E and H strongly fluctuated due to surface roughness both (Fig. 2g), and at higher depths the side effects of the epoxy could not be excluded. All statistical analyses were performed with JMP^®^ Pro, Version 14 (SAS Institute Inc., Cary, NC, 1989–2007), calculating mean values and standard deviations summarizing the data of all measured cusps per tooth type of all analysed specimens. This was done for each species. Shapiro–Wilk-W-test for testing of normality and one-way ANOVA followed by a Tukey–Kramer test for detecting homogeneous groups with connecting letters report were carried out afterwards. Mechanical properties were compared between the preferred feeding substrates (i), all central, lateral, and marginal tooth cusps (ii), within each species (iii), between the species (iv). Additionally, a two-way ANOVA for determining the influence of the feeding substrate, the tooth type and the interaction of both parameters was carried out.

For scanning electron microscopy (SEM) one radula per species (Additional file 1: Table S1) was extracted, digested with proteinase K according to the protocol of [147], cleaned for a few seconds in an ultrasonic bath, mounted on an aluminium stub, coated with carbon and visualized either with a SEM Zeiss LEO 1525 (One Zeiss Drive, Thornwood, NY) or a Tabletop SEM TM4000Plus (Hitachi, Tokyo, Japan) (see Figs. 3, 4, 5, 6, 7 and 8 for SEM images).

To establish a hypothesis on the evolutionary history of the African paludomids in the context of trophic specialisation based on biomechanical properties, we used all available DNA sequences of tested species, here sequences of the mitochondrial 16S rRNA (16S) and the cytochrome c oxidase subunit I (COI) gene from previous studies [42, 85] (see Additional file 1: Table S1); additionally, *Melanoides tuberculata* and *Paludomus siamensis* sequences were used as outgroup. Sequences were aligned with MAFFT 7 [148] employing the Q-INS-I algorithm, the 1PAM/κ = 2 option for the scoring matrix for nucleotide sequences and otherwise default settings. Bayesian inference with MrBayes 3.2.6 [149] was used to reconstruct phylogenetic relationship. PartitionFinder 2.1.1 [150] was used to select best-fitting models and a suitable partitioning strategy for the Bayesian inference based on the Bayesian information criterion. The DNA sequences were initially divided into four partitions: the first, second and third codon positions of COI and 16S. An exhaustive search with PartitionFinder was conducted allowing for separate estimation of branch lengths for each partition. The models were limited to those available in MrBayes. Metropolis coupled Markov chain Monte Carlo (MC^3^) searches were run with four chains in two separate runs for 50,000,000 generations with default priors, trees sampled every 1000 generations and separate estimation of parameters for individual partitions under default heating using best-fit models as suggested by PartitionFinder (first plus second codon positions of COI: GTR + I + G; third codon positions of COI: HKY + G; 16S: GTR + I + G). Diagnostic tools provided in MrBayes were used to ensure that the MC^3^ searches had reached stationarity and convergence. The first 5,000,000 generations of each run were discarded as burn-in.

Changes of Young’s modulus over the phylogeny (excl. outgroups) for the three different tooth types were visualized using continuous character mapping. Ancestral states [see also 145] for internal nodes were estimated using a maximum likelihood approach along with interpolating the states along the branches of the tree following an idea from [151] as implemented in phytools [152, 153]. To trace the adaptation to different feeding substrates (soft, solid, mixed) in a maximum likelihood setting, we used ape [154] assuming the one-parameter equal rates model to specify the transition probabilities between the states of the discrete character.

For some species, *Bridouxia praeclara*, *B. rotundata*, *Paramelania crassigranulata*, *Mysorelloides multisulcata*, no molecular information could be obtained from various previous approaches. These taxa were placed tentatively in the resulting phylogeny as suggested by [44, 155] who compared internal and external morphological characters and identified synapomorphies. This resulted here in a systematization [see 156 for further details of this term].

The information on the preferred feeding substrate is based on the relevant literature [44, 79, 120, 139] supplemented by notes from the collectors of individual samples in the field (Heinz Büscher, Matthias Glaubrecht).

## Supplementary Information

**Additional file 1: Table S1.** List of specimens, collection numbers, localities, and preparation techniques; **Fig. S1** und **Tables S2**, **S3** Results of 2-way ANOVA for E and H.

## Data Availability

The datasets used and/or analysed during the current study available from the corresponding author on reasonable request.

## Abbreviations

16S: Mitochondrial 16S rRNA
ANOVA: Analysis of variance
COI: Cytochrome *c* oxidase subunit I gene
E: Young's modulus
H: Hardness
IRSNB: Royal Belgian Institute of Natural Sciences, Bruxelles, Belgium
MRAC: Musée royal de l'Afrique centrale, Tervuren, Belgium
N: Quantity
SEM: Scanning Electron Microscope
ZMB: Museum für Naturkunde Berlin, Germany
ZMH: Zoologisches Museum Hamburg (Center of Natural History CeNak), Germany
