# Supplementary material for: Trophic specialisation reflected by radular tooth material properties in an “ancient” Lake Tanganyikan gastropod species flock
Source: BMC Ecol Evol. 2021 Mar 3;21:35. doi: 10.1186/s12862-021-01754-4 (PMC7931582; doi:10.1186/s12862-021-01754-4)
Supplement: Supplementary file 1 — Additional file 1: Table S1. List of specimens, collection numbers, localities, and preparation techniques; Fig. S1 und Tables S2, S3 Results of 2-way ANOVA for E and H. [file 12862_2021_1754_MOESM1_ESM.docx]

**Table S1.** Specimens, collection number, locality, and preparation technique (SEM, nanoindentation, molecular, GenBank accession numbers of COI and 16S sequences). IRSNB = Institut royal des Sciences naturelles de Belgique, Bruxelles, Belgium; MRAC = Musée royal de l'Afrique centrale, Tervuren, Belgium; SEM = Scanning Electron Microscope; ZMB = Museum für Naturkunde Berlin, Germany; ZMH = Zoologisches Museum Hamburg (Center of Natural History CeNak), Germany.

| Groups | Species | Collection number | Locality | Preparation or method | Molecular data, GenBank accessions | | |
| --- | --- | --- | --- | --- | --- | --- | --- |
|  |  |  |  |  | COI | 16S | Reference of molecular data |
| Group 1 | *Bridouxia grandidieriana* (Bourguignat, 1885) | ZMH 119367/999_1–9 | Zambia, 08°43'25''S, 31°09'00''E | Nanoindentation |  |  |  |
|  |  | ZMB 220.009x | Zambia, Mpulungu Field Station | Molecular | AY456533 | AY456587 | [42] |
|  |  | ZMB 220.139_4 | Burundi | SEM |  |  |  |
|  | *Bridouxia ponsonbyi* (Smith, 1889) | ZMB 220.137_1 | Tanzania, Kigoma | SEM |  |  |  |
|  |  | ZMB 220.137_2–6, _9 |  | Nanoindentation |  |  |  |
|  |  | ZMB 220.137 |  | Molecular |  | Will be added after acceptance | This study |
|  | *Bridouxia praeclara* Bourguignat, 1885 | ZMB 220.010_1–4 | Zambia, 08°45'258'S, 31°05'116'E | Nanoindentation |  |  |  |
|  |  | ZMB 220.063_2 | Tanzania, Kigoma | SEM |  |  |  |
|  | *Bridouxia rotundata* (Smith, 1904) | ZMB 220.063_1 | Tanzania, Kigoma | SEM |  |  |  |
|  |  | ZMB 220.063_3–8 |  | Nanoindentation |  |  |  |
|  | *Cleopatra johnstoni* Smith, 1893 | ZMB 220.102_1 | Zambia, 09°20'866'S, 28°43’886'E | SEM |  |  |  |
|  |  | ZMB 220.102b_1–8 |  | Nanoindentation |  |  |  |
|  |  | ZMB 220.102a |  | Molecular | AY456536 | AY456590 | [42] |
|  | *Leloupiella minima* (Smith, 1908) | ZMB 220.008_1 | Zambia, 08°45'258'S, 31°05'116'E | Nanoindentation |  |  |  |
|  |  | ZMB 220.008x |  | Molecular | AY456576 | AY456630 | [42] |
|  |  | ZMB 220.135 | Tanzania, Kigoma | SEM |  |  |  |
|  |  | ZMH 150017/999_1–4 | Zambia, 08°42'10''S, 30°55’21''E | Nanoindentation |  |  |  |
|  | *Reymondia horei* (Smith, 1880a) | ZMB 220.147_1 | Tanzania, Kigoma | SEM |  |  |  |
|  |  | ZMB 220.147_10 |  | Nanoindentation |  |  |  |
|  |  | ZMB 220.013a | Zambia, Kasenga Point | Molecular | AY456568 | AY456622 | [42] |
|  |  | ZMH 150007/999_1–5 | Zambia, 08°37'23''S, 31°12’01''E | Nanoindentation |  |  |  |
|  | *Spekia zonata* (Woodward, 1859) | ZMB 220.077_1–2 | Zambia, 08°45'547'S, 31°05'825'E | Nanoindentation, data taken from [124] |  |  |  |
|  |  | ZMB 220.143_1–4 | Tanzania, Kigoma | Nanoindentation, data taken from [124] |  |  |  |
|  |  | ZMH 150008/999_1 | Zambia, 08°36'31''S, 31°11’42''E | Nanoindentation, data taken from [124] |  |  |  |
|  |  | ZMH 150008/999_2 |  | SEM |  |  |  |
|  |  | ZMB 220.026a | Zambia, Kumbula Island | Molecular | AY456569 | AY456623 | [42] |
| Group 2 | *Anceya giraudi* Bourguignat, 1885 | ZMB 220.132 | Tanzania, Kigoma | SEM |  |  |  |
|  |  | ZMB 220.000b | Zambia, Kasenga Point | Molecular | AY456529 | AY456529 | [42] |
|  |  | ZMH 150015/999_1–5 | Zambia, 08°35'45''S, 30°48’27''E | Nanoindentation |  |  |  |
|  | *Martelia tanganyicensis* Dautzenberg, 1907 | ZMB 220.133_1 | Tanzania, Kigoma | SEM |  |  |  |
|  |  | ZMB 220.133_2–5 |  | Nanoindentation |  |  |  |
|  |  | ZMB 220.006x | Zambia, Kumbula Island | Molecular | AY456578 | AY456632 | [42] |
|  | *Stanleya neritinoides* (Smith, 1880a) | MRAC without number | “Lake Tanganyika” | SEM |  |  |  |
|  |  |  |  | Molecular | AY213146 |  | [85] |
|  |  | ZMB 102.624_1–4 | Tanzania, Kiranda | Nanoindentation |  |  |  |
|  | *Syrnolopsis lacustris* Smith, 1880a | ZMB 220.045_1–9 | Zambia, 08°42'887'S, 31°08’476'E | Nanoindentation |  |  |  |
|  |  | ZMB 220.131 | Tanzania, Kigoma | SEM |  |  |  |
|  |  | ZMB 220.046a | Zambia, Mpulungu Field Station | Molecular | AY456574 | AY456628 | [42] |
|  | *Tanganyicia rufofilosa* (Smith 1880a) | ZMH 150009/999_1–7 | Zambia, 08°46'48''S, 31°00'22''E | Nanoindentation |  |  |  |
|  |  |  | “Lake Tanganyika” | SEM |  |  |  |
|  |  | ZMB 220.039a |  | Molecular | AY456580 | AY456634 | [42] |
| Group 3 | *Chytra kirki* (Smith, 1880a) | IRSNB no. 63 | Tanzania, Malagarasi delta | SEM |  |  |  |
|  |  | ZMB 220.155_1–3 | Russago | Nanoindentation |  |  |  |
|  |  |  | Zambia, Wonzye Point | Molecular | AY213142 |  | [85] |
|  | *Limnotrochus thomsoni* Smith, 1880a | ZMB 107.102 | Zambia, 08°47'52''S, 31°01'11''E | SEM |  |  |  |
|  |  | ZMB 220.038a | Zambia, Kumbula Island | Molecular | AY456558 | AY456612 | [42] |
|  |  | ZMH 119371/999_1–8 | Zambia, 08°47'50''S, 31°01'02''E | Nanoindentation |  |  |  |
|  | *Mysorelloides multisulcata* (Bourguignat, 1888) | IRSNB no. 126 | Tanzania, Karema | SEM |  |  |  |
|  |  | ZMH without number_1–2 | Zambia, 08°43'25''S, 31°09'00''E | Nanoindentation |  |  |  |
|  | *Paramelania crassigranulata* (Smith, 1881) | ZMB 220.037_1 | Tanzania, Kigoma | SEM |  |  |  |
|  |  | ZMB 220.037_2–4 |  | Nanoindentation |  |  |  |
|  | *Paramelania damoni* (Smith, 1881) | ZMH without number | “Lake Tanganyika” | SEM |  |  |  |
|  |  |  |  | Molecular | AY213152 |  | [85] |
|  |  | ZMH 150023/999_1–4 | Zambia, 08°34'09''S, 31°45'02''E | Nanoindentation |  |  |  |
|  | *Paramelania iridescens* (Moore, 1898c) | ZMB 220.053 | Burundi, Busumbura | SEM |  |  |  |
|  |  | ZMB 220.099_1–4 | Zambia, 08°41'700'S, 31°08'952'E | Nanoindentation |  |  |  |
|  |  | ZMB 220.099a |  | Molecular | AY456566 | AY456620 | [42] |
|  | *Tiphobia horei* Smith, 1880b | ZMB 220.095_1–2 | Zambia, 08°41'700'S, 31°08'952'E | Nanoindentation |  |  |  |
|  |  | ZMB 220.095 |  | Molecular | AY456582 | AY456636 | [42] |
| Group 4 | *Lavigeria grandis* (Smith, 1881) | ZMB 220.018a | Zambia, 08°42'887'S, 31°08'476'E | Molecular | AY456538 | AY456592 | [42] |
|  |  | ZMB 220.018_1 |  | Nanoindentation |  |  |  |
|  |  | ZMH 150020/999_1–5 | Zambia, 08°43'25''S, 31°09'00''E | Nanoindentation |  |  |  |
|  |  | ZMH 154657/999 | Zambia, Mibwebwe | SEM |  |  |  |
|  | *Lavigeria livingstoniana* (Bourguignat, 1885) | ZMB 220.117_1–5 | Tanzania, Kigoma | SEM |  |  |  |
|  |  | ZMB 107.097 | Tanzania, Utinta | Molecular | Will be added after acceptance | Will be added after acceptance | This study |
|  | *Lavigeria nassa* (Woodward, 1859) | ZMB 220.074 | Zambia, 08°45'547'S, 31°05'825'E | SEM |  |  |  |
|  |  | ZMH 119369/999_1–9 | Zambia, 08°29'23''S, 30°28'46''E | Nanoindentation |  |  |  |
|  |  | ZMB 220.019a | Zambia, Kasenga Point | Molecular | AY456548 | AY456602 | [42] |
|  | *Lavigeria spinulosa* (Bourguignat, 1885) | ZMB 220.051 | Zambia, 08°42'258'S, 31°05’116'E | SEM |  |  |  |
|  |  | ZMB 220.014a | Zambia, Kumbula Island | Molecular | AY456555 | AY456609 | [42] |
|  |  | ZMH 150012/999_1–6 | Zambia, 08°42'10''S, 30°55’21''E | Nanoindentation |  |  |  |
| Outgroup | *Paludomus siamensis* Blanford, 1903 | ZMB 200.231 |  | Molecular | AY456560 | AY456614 | [42] |
| Outgroup | *Melanoides tuberculata* (Müller, 1774) | ZMB 220.060 | Malawi | Molecular | AY456562 | AY456616 | [42] |

**Figure S1.** Least square mean plots of 2-way ANOVA for E (*left side*) and H (*right side*).


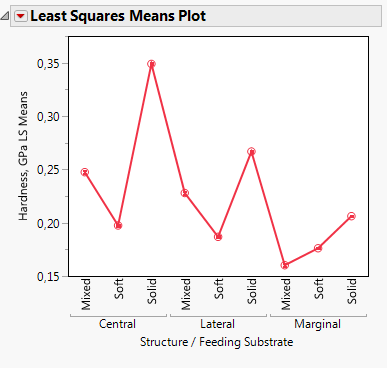

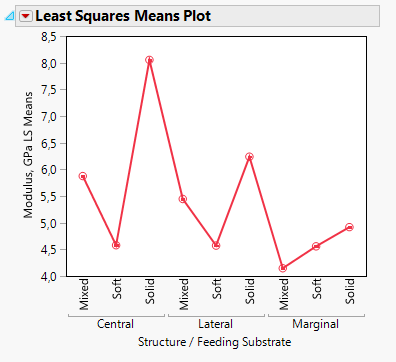


**Table S2.** 2-way ANOVA table and the effect tests with the interaction terms for Modulus.

| **Analysis of Variance for Modulus** | | | | |  |
| --- | --- | --- | --- | --- | --- |
| **Source** | **DF** | **Sum of**  **Squares** | **Mean Square** | **F Ratio** | **Prob > F** |
| Model | 8 | 464645.43 | 58080.7 | 132707.8 | <.0001* |
| Error | 325214 | 142332.65 | 0.437658 |  |  |
| C. Total | 325222 | 606978.07 |  |  |  |
| **Effect Tests for Modulus** | | | | | |
| **Source** | **Nparm** | **DF** | **Sum of**  **Squares** | **F Ratio** | **Prob > F** |
| Tooth Structure | 2 | 2 | 118383.59 | 135246.6 | <.0001* |
| Feeding Substrate | 2 | 2 | 216669.39 | 247532.5 | <.0001* |
| Tooth Structure*Feeding Substrate | 4 | 4 | 113973.26 | 65104.01 | <.0001* |

**Table S3.** 2-way ANOVA table and the effect tests with the interaction terms for Hardness.

| **Analysis of Variance for Hardness** | | | | |  |
| --- | --- | --- | --- | --- | --- |
| **Source** | **DF** | **Sum of**  **Squares** | **Mean Square** | **F Ratio** | **Prob > F** |
| Model | 8 | 1051.0364 | 131.380 | 17617.55 | <.0001* |
| Error | 327495 | 2442.2318 | 0.007457 |  |  |
| C. Total | 327503 | 3493.2682 |  |  |  |
| **Effect Tests for Hardness** | | | | |  |
| **Source** | **Nparm** | **DF** | **Sum of**  **Squares** | **F Ratio** | **Prob > F** |
| Tooth Structure | 2 | 2 | 316.14352 | 21196.89 | <.0001* |
| Feeding Substrate | 2 | 2 | 496.44241 | 33285.62 | <.0001* |
| Tooth Structure*Feeding Substrate | 4 | 4 | 176.81568 | 5927.596 | <.0001* |
